# The Singlet–Triplet Gap of Cyclobutadiene: The CIPSI-Driven CC(*P*;*Q*) Study

**DOI:** 10.1021/acs.jpca.5c07572

**Published:** 2025-12-09

**Authors:** Swati S. Priyadarsini, Karthik Gururangan, Jun Shen, Piotr Piecuch

**Affiliations:** † Department of Chemistry, 3078 Michigan State University, East Lansing, Michigan 48824, United States; ‡ Department of Physics and Astronomy, Michigan State University, East Lansing, Michigan 48824, United States

## Abstract

An accurate determination of singlet–triplet gaps in biradicals, including cyclobutadiene in the automerization barrier region where one has to balance the substantial nondynamical many-electron correlation effects characterizing the singlet ground state with the predominantly dynamical correlations of the lowest-energy triplet, remains a challenge for many quantum chemistry methods. High-level coupled-cluster (CC) approaches, such as the CC method with a full treatment of singly, doubly, and triply excited clusters (CCSDT), are often capable of providing reliable results, but routine application of such methods is hindered by their high computational costs. We have recently proposed a practical alternative to converging the CCSDT energetics at small fractions of the computational effort, even when electron correlations become stronger and connected triply excited clusters are larger and nonperturbative, by merging the CC­(*P*;*Q*) moment expansions with the selected configuration interaction methodology abbreviated as CIPSI. We demonstrate that one can accurately approximate the highly accurate CCSDT potential surfaces characterizing the lowest singlet and triplet states of cyclobutadiene along the automerization coordinate and the gap between them using tiny fractions of triply excited cluster amplitudes identified with the help of relatively inexpensive CIPSI Hamiltonian diagonalizations.

## Graphical abstract

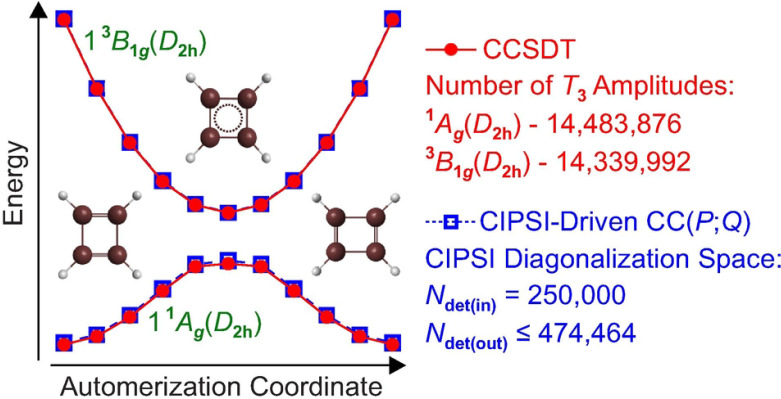

## 1 Introduction

Biradicals play a key role in chemistry as reaction intermediates in thermal and photochemical pathways^1–7^ as well as functional materials used in molecular magnets,^8–10^ battery electrodes,^11^ and organic photovoltaics.^12–15^ An important quantity characterizing the electronic structure of biradicals, especially in the context of designing molecules for magnetic, electrochemical, and photovoltaic applications, is the energy gap Δ*E*_S–T_ separating the lowest-lying singlet and triplet states (throughout this work, we define Δ*E*_S–T_ as *E*_S_ – *E*_T_, where *E*_S_ and *E*_T_ are the electronic energies of the relevant singlet and triplet states, i.e., when the singlet is lower than the triplet, Δ*E*_S–T_ < 0). Accurate computational determination of the Δ*E*_S–T_ values in biradicals remains, however, a difficult task because it requires balancing the strong nondynamical many-electron correlation effects characterizing the low-spin singlet states with the predominantly dynamical correlations associated with their high-spin triplet counterparts.^16–34^

This challenge is exemplified by the cyclobutadiene molecule, which is the focus of the present study and which has fascinated experimental and theoretical chemists for decades with questions surrounding its low-lying electronic states, antiaromaticity, and reactivity in cycloaddition and isomerization reactions. In the *D*_4h_-symmetric square structure corresponding to the barrier along the automerization coordinate or the minimum on the lowest triplet potential, cyclobutadiene is a biradical with its four valence π orbitals arranged in a network consisting of the nondegenerate *a*_2*u*_ orbital, the doubly degenerate *e*_*g*_ shell, and the nondegenerate *b*_1*u*_ orbital. The distribution of two of the four valence electrons among the pair of degenerate frontier *e*_*g*_ orbitals gives rise to three singlet states of the *B*_1*g*_(*D*_4h_), *A*_1*g*_(*D*_4h_), and *B*_2*g*_(*D*_4h_) symmetries and a *A*_2*g*_(*D*_4h_)-symmetric triplet state, all involving the doubly occupied *a*_2*u*_ and unoccupied *b*_1*u*_ orbitals and the partially occupied *e*_*g*_ shell in their zeroth-order description.^30,35–41^ As shown in the early *ab initio* calculations,^35,36,42–44^ and as confirmed in many subsequent theoretical studies, such as those reported in refs 20, 26–28, 30–34, and 37–41 , the lowest singlet of the *B*_1*g*_(*D*_4h_) symmetry, which has a substantial multiconfigurational character, is the ground state, whereas the predominantly single-reference *A*_2*g*_(*D*_4h_)-symmetric triplet, in violation of Hund’s rule, is the first excited state. To be more specific, if one orients cyclobutadiene such that the two *C*_2_ axes bisect the carbon–carbon bonds, which is a convention adopted in the present study, the ^1^ *B*_1*g*_(*D*_4h_) ground state of the square structure is dominated by two closed-shell determinants in which one of the two degenerate *e*_*g*_ orbitals is occupied by two electrons and the other one is empty (see, e.g., refs 37, 39, 41, and 44 ). This should be contrasted with the lowest-energy ^3^ *A*_2*g*_(*D*_4h_) state, which is characterized by a single occupancy of each of the *e*_*g*_ orbitals.

While the lowest ^3^ *A*_2*g*_(*D*_4h_) state is stable in the square geometry, which is a minimum on the corresponding triplet surface, the ^1^ *B*_1*g*_(*D*_4h_) ground state is unstable with respect to the rectangular distortion of the carbon–carbon bonds that lifts the degeneracy of the valence *e*_*g*_ orbitals and lowers its total electronic energy due to the pseudo-Jahn–Teller effect. This results in the formation of the *D*_2h_-symmetric rectangular species characterized by the closed-shell, predominantly single-determinantal, ^1^ *A*_*g*_(*D*_2h_) ground state, which represents a minimum on the lowest-energy singlet potential (see, e.g., refs 30, 31, 33, 34, 37–39, 41, and 44 ). The distortion of the multiconfigurational ^1^ *B*_1*g*_(*D*_4h_) state into the ^1^ *A*_*g*_(*D*_2h_) state coincides with the automerization coordinate in cyclobutadiene, which describes the conversion of the rectangular, *D*_2h_-symmetric, closed-shell reactant (R) into the equivalent product conformer by passing through the square, *D*_4h_-symmetric, biradical transition state (TS). Obtaining an accurate description of the potential energy curves (PECs) characterizing the lowest singlet [^1^ *A*_*g*_(*D*_2h_)] and triplet [^3^ *B*_1*g*_(*D*_2h_)] states of cyclobutadiene along its *D*_2h_-symmetric automerization coordinate, and the gap between them, particularly in the neighborhood of the square biradical species, remains a significant challenge for modern *ab initio* techniques as it requires a high-level treatment of many-electron correlation effects in order to accurately capture and balance the strong nondynamical correlations associated with the multiconfigurational singlet state with the largely dynamical correlations dominating the triplet state.

A traditional way of addressing this and similar challenges is to use multireference approaches,^45–52^ but in this work we focus on the single-reference coupled-cluster (CC) methodology,^53–59^ which employs the exponential wave function ansatz^60,61^

1 $$\left|\Psi \right\rangle =e^{T}|\Phi \rangle ,$$

where |Φ⟩ is the *N*-electron reference determinant that serves as a Fermi vacuum and *T* = ∑_*n*=1_^*N*^ *T*_*n*_ is the cluster operator, with *T*_*n*_ designating the *n*-body component of *T* responsible for generating connected *n*-particle–*n*-hole (*n*p-*n*h) excitations out of |Φ⟩. It is well established that as long as the number of strongly correlated electrons is not too large, the standard hierarchy of CC approximations, including the CC method with singles and doubles (CCSD),^62–65^ obtained by truncating *T* at *T*_2_, the CC approach with singles, doubles, and triples (CCSDT),^66–69^ in which *T* is truncated at *T*_3_, the CC method with singles, doubles, triples, and quadruples (CCSDTQ),^70–73^ where *T* is truncated at *T*_4_, and so on, rapidly converges to the exact, full configuration interaction (CI) limit. As a result, the single-reference CC approaches with a full treatment of higher-rank *T*_*n*_ clusters with *n* > 2, such as CCSDT or CCSDTQ, are often capable of accurately describing multireference situations, including substantial bond rearrangements in the course of chemical reactions and, what is especially important for this study, singlet–triplet gaps in biradical species, by capturing the relevant dynamical and nondynamical correlation effects via particle–hole excitations from a single determinant, without having to involve genuine multireference concepts.

In particular, the calculations reported in refs 32, 38 , and^41^ show that the full treatment of *T*_1_, *T*_2_, and *T*_3_ clusters provided by CCSDT offers a highly accurate description of the total electronic energies of the lowest ^1^ *B*_1*g*_(*D*_4h_) and ^3^ *A*_2*g*_(*D*_4h_) states of the square cyclobutadiene and the gap between them. As demonstrated in Figure 1, this remains true when examining the PECs characterizing the lowest-energy singlet and triplet states of cyclobutadiene along the entire *D*_2h_-symmetric automerization reaction path. In determining the lowest-energy ^1^ *A*_*g*_(*D*_2h_) and ^3^ *B*_1*g*_(*D*_2h_) PECs shown in Figure 1 (for information about the electronic structure software used in our calculations, see Section 2), we followed the procedure described in ref 40 in which one constructs an approximate, *D*_2h_-symmetric, one-dimensional automerization pathway connecting the rectangular minima on the lowest singlet potential via the square TS species by linearly interpolating the carbon–carbon bond distances in cyclobutadiene using the formula

2 $$l_{i}\left(\lambda \right)=\left(1-\lambda \right)l_{i}\left(R\right)+\lambda l_{i}\left(\mathrm{TS}\right),,i=1,2,$$

where $l_{1}$ and $l_{2}$ are the C–C distances depicted in Figure 1 and $l_{i}\left(R\right)$ and $l_{i}\left(\mathrm{TS}\right)$ are the carbon–carbon bond lengths characterizing the R and TS structures optimized (along with the C–H distances and H–C–C bond angles) in ref 40 with the multireference average quadratic CC (MR-AQCC) approach.^74,75^ The dimensionless parameter λ defining the automerization coordinate varies between 0, corresponding to the R species, and 1, corresponding to the TS structure, going back to 0 (after replacing $l_{1}$ by $l_{2}$ and *vice versa*) when the automerization product equivalent to the R species is reached. In the absence of information about the C–H bond lengths and H–C–C bond angles characterizing the intermediate λ = 0.2, 0.4, 0.6, and 0.8 geometries in ref 40, in determining the lowest ^1^ *A*_*g*_(*D*_2h_) and ^3^ *B*_1*g*_(*D*_2h_) states of these structures, we fixed the C–H distances and H–C–C angles at their values corresponding to the R species.

**Figure 1:**
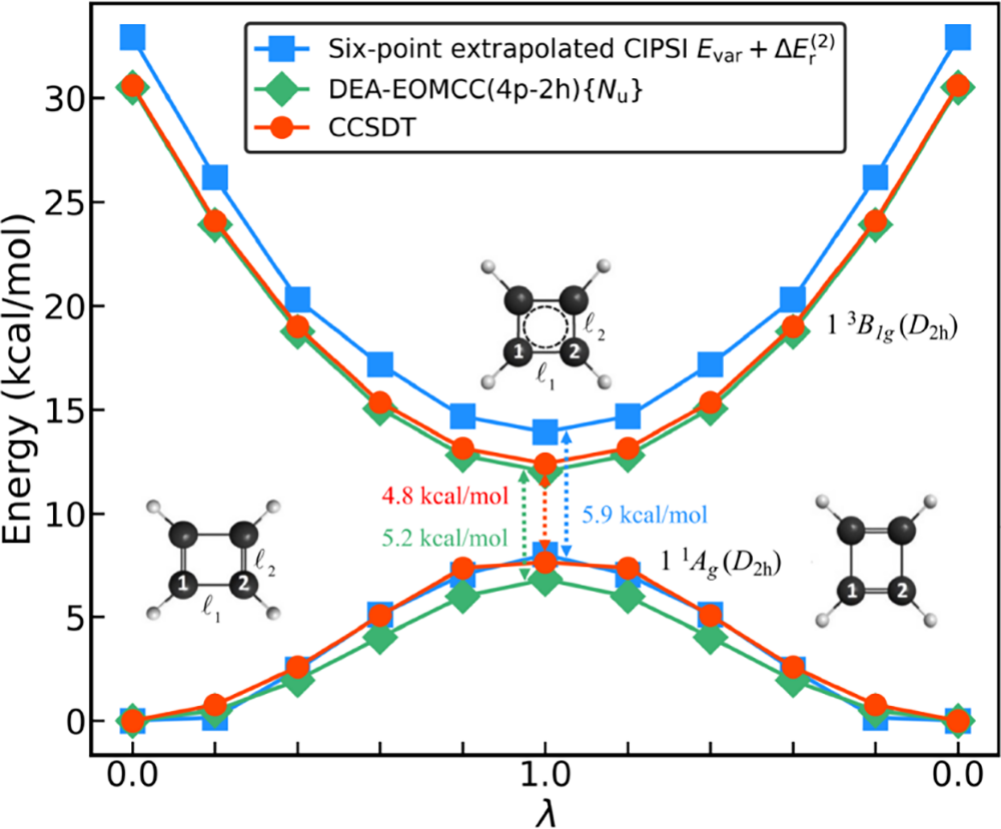
The PECs (in kcal/mol) characterizing the lowest-energy singlet and triplet states of cyclobutadiene along the *D*_2h_-symmetric automerization pathway, defined using the interpolation formula given by eq 2 and parametrized by the dimensionless variable λ, resulting from the full CCSDT (red solid circles and lines), active-space DEA-EOMCC­(4p-2h)­{*N*_u_} (green solid diamonds and lines), and perturbatively corrected and extrapolated CIPSI (blue solid squares and lines) calculations using the cc-pVDZ basis set described in the main text. For each of the three methods, the energy of the singlet ground state at the reactant (R, λ = 0) geometry is set to 0. The numbers in the middle, colored in the same way as the corresponding PECs, are the unsigned values of the singlet–triplet gaps determined at the λ = 1 TS structure.

As shown in Figure 1, the lowest singlet and triplet PECs computed as functions of the *D*_2h_-symmetric automerization coordinate λ with full CCSDT are in very good agreement with their counterparts obtained with the CI method using perturbative selection made iteratively,^76–78^ abbreviated as CIPSI, extrapolated to the full CI limit (see Section 2 for further details). The CCSDT energies are also very close to those determined using the double electron-attachment (DEA) equation-of-motion (EOM) CC methodology^26,29,30,79–82^ with a full treatment of 2p and 3p-1h and an active-space treatment of 4p-2h correlations on top of the CCSD description of the underlying closed-shell (C_4_H_4_)^2+^ core,^26,29,81,82^ denoted as DEA-EOMCC­(4p-2h)­{*N*_u_}, where *N*_u_ designates the number of active unoccupied orbitals of (C_4_H_4_)^2+^ included in the calculations to capture the leading 4p-2h effects in the target cyclobutadiene species (to accurately describe the 4p-2h effects associated with the valence orbitals of cyclobutadiene that correlate with the *e*_*g*_ and *b*_1*u*_ shells of the square TS structure, we set *N*_u_ to 3). To illustrate the agreement between full CCSDT, perturbatively corrected and extrapolated CIPSI, and DEA-EOMCC­(4p-2h)­{*N*_u_}, which are three independent *ab initio* methodologies, we compare the vertical Δ*E*_S–T_ values at the R and TS geometries. When using the cc-pVDZ^83^ basis set, employed in the calculations reported in Figure 1 and in most of the computations discussed in the rest of this article, the CCSDT value of Δ*E*_S–T_ at the TS geometry is −4.8 kcal/mol. This is very close to −5.2 kcal/mol resulting from the state-of-the-art DEA-EOMCC­(4p-2h)­{*N*_u_} calculations and −5.9 kcal/mol obtained with CIPSI. The CCSDT, DEA-EOMCC­(4p-2h)­{*N*_u_}, and perturbatively corrected and extrapolated CIPSI values of Δ*E*_S–T_ at the R geometry are −30.6, −30.5, and −32.9 kcal/mol, respectively, again in good agreement with one another.

Given the high accuracy of the lowest singlet and triplet potential surfaces of cyclobutadiene and Δ*E*_S–T_ values along the *D*_2h_-symmetric automerization pathway offered by full CCSDT, it may be tempting to turn to the approximate treatments of *T*_3_ correlations that replace the expensive iterative $N^{8}$ computational steps of CCSDT, where N is a measure of the system size, by the more practical $N^{6}$ operations of CCSD combined with the noniterative $N^{7}$ steps needed to correct the CCSD energetics for the leading *T*_3_ correlations, as in the widely used CCSD­(T) approach^84,85^ or its more robust completely renormalized (CR) CC counterpart abbreviated as CR-CC­(2,3).^17,86–88^ Unfortunately, neither CCSD­(T) and CR-CC­(2,3) nor any of the other noniterative triples corrections to CCSD, such as CCSD­(T)_Λ_,^89–91^ CCSD(2)_T_,^92–95^ CR-CCSD­(T)^96–99^ and its locally renormalized extension,^100^ Λ-CCSD­(T),^101,102^ and CCSD­(T–*n*),^103,104^ are capable of providing accurate results when the coupling of the lower-order *T*_1_ and *T*_2_ components of the cluster operator with their higher-rank *T*_3_ counterpart becomes large. For example, even the most robust triples correction to CCSD defining CR-CC­(2,3), which improves CCSD­(T) and other similar approaches in situations involving electronic quasi-degeneracies, such as those present in single bond breaking,^17,86–88,105–108^ struggles in describing the PEC of the lowest ^1^ *A*_*g*_(*D*_2h_) state of cyclobutadiene in the neighborhood of the automerization barrier region, where *T*_3_ clusters become large, nonperturbative, and strongly coupled to *T*_1_ and *T*_2_.^109^ As a result, as shown in ref 32 and this study, the CR-CC­(2,3) Δ*E*_S–T_ value at the singlet TS structure, of 4.4 kcal/mol when the cc-pVDZ basis is used, is in large error (including incorrect sign) relative to its CCSDT −4.8 kcal/mol counterpart. CCSD­(T) gives 3.9 kcal/mol, which is similarly inaccurate. This is because neither CR-CC­(2,3) nor CCSD­(T), nor any other noniterative triples correction to CCSD, can correctly describe *T*_3_ contributions to Δ*E*_S–T_ that at the square TS geometry of cyclobutadiene are a few times larger, in absolute value, than the CCSDT singlet–triplet gap itself. For example, the *T*_3_ effects on Δ*E*_S–T_ at the TS structure, estimated by subtracting the singlet–triplet gap obtained in the CCSD/cc-pVDZ calculations from its CCSDT/cc-pVDZ counterpart, are −15.1 kcal/mol, i.e., they are more than three times larger than the value of Δ*E*_S–T_ obtained in the CCSDT/cc-pVDZ calculations. The fact that the *T*_3_ contributions to the singlet–triplet gap of cyclobutadiene in the vicinity of the automerization barrier region become so massive and difficult to describe by the noniterative triples corrections to CCSD is closely related to the dramatic increase of *T*_1_, *T*_2_, and *T*_3_ cluster amplitudes characterizing the lowest-energy ^1^ *A*_*g*_(*D*_2h_) state, especially the amplitudes defining *T*_2_ and *T*_3_ operators that engage valence orbitals around the Fermi level, reflecting on the increasingly strongly correlated character of this state as one transitions from the R to TS structures. For example, the largest *T*_1_, *T*_2_, and *T*_3_ amplitudes obtained in the CCSDT/cc-pVDZ calculations for the *A*_*g*_(*D*_2h_)-symmetric singlet ground state of cyclobutadiene at its R geometry are −0.032682, −0.206225, and 0.003414, respectively. At the TS geometry, they become −0.054928, −0.895785, and 0.014836, respectively, i.e., we observe a 4-fold increase in the largest *T*_2_ and *T*_3_ amplitudes compared to the R structure. This should be contrasted with the behavior of the lowest-energy triplet state, where the largest *T*_1_, *T*_2_, and *T*_3_ cluster amplitudes resulting from the CCSDT/cc-pVDZ computations remain relatively small and barely change when the R → TS (λ = 0 → 1) geometrical transformation is examined. They are 0.031220 for *T*_1_, −0.163848 for *T*_2_, and 0.003285 for *T*_3_ at λ = 0 and 0.030476, −0.137065, and 0.002555, respectively, when λ becomes 1. It is this drastically different behavior of the lowest ^1^ *A*_*g*_(*D*_2h_) and ^3^ *B*_1*g*_(*D*_2h_) states of cyclobutadiene and the rapid growth of the cluster amplitudes characterizing the former state that engage valence orbitals, especially those associated with *T*_2_ and *T*_3_, as the automerization barrier region is approached, which result in failures of methods such as CCSD­(T) and CR-CC­(2,3) in describing the corresponding singlet–triplet gap.

Problems with applying noniterative corrections to CCSD in situations where *T*_*n*_ components with *n* > 2, such as *T*_3_, are not only large and nonperturbative but also strongly coupled to their lower-rank *T*_1_ and *T*_2_ counterparts have motivated us to develop the generalization of the biorthogonal moment expansions, which in the past resulted in the CR-CC approaches, such as CR-CC­(2,3) and its excited-state and higher-order extensions,^17,86–88,106,110–116^ to unconventional truncations in the cluster and EOMCC^117–120^ excitation operators, designated as CC­(*P*;*Q*).^22,32,105,109,115,116,121–127^ By incorporating the dominant contributions to the higher-than-two-body clusters into the iterative steps, so that *T*_1_ and *T*_2_ amplitudes can relax compared to their CCSD values when *T*_*n*_ components with *n* > 2 become more substantial, and correcting the results for the remaining many-electron correlation effects of interest using suitably defined moment expansions, the CC­(*P*;*Q*) formalism provides us with the opportunity to converge or accurately approximate the parent high-level CCSDT, CCSDTQ, and similar energetics at small fractions of the computational costs, even when noniterative corrections to CCSD fail or struggle. Focusing on full CCSDT, which provides the parent data for the lowest singlet and triplet PECs of cyclobutadiene examined in this work, a few different CC­(*P*;*Q*) approaches designed to converge CCSDT energetics have been developed. In the initial, active-orbital-based, variant of CC­(*P*;*Q*), abbreviated as CC­(t;3), which is part of the larger CC­(t;3), CC­(t,q;3), CC­(t,q;3,4), CC­(q;4), etc. hierarchy,^22,105,109,115,116,121,127^ the leading *T*_3_ amplitudes that enter the iterative steps preceding the determination of the CC­(*P*;*Q*) corrections are obtained using the active-space CCSDt approach.^73,128–136^ In the more black-box semistochastic^32,122–124^ and CIPSI-driven CC­(*P*;*Q*)^125^ approaches aimed at converging CCSDT, the dominant triply excited cluster amplitudes are identified with the help of CI^137–141^ or CC^142–145^ Quantum Monte Carlo wave function propagations in the many-electron Hilbert space, in the former case, and the sequences of Hamiltonian diagonalizations constructed in the CIPSI algorithm^76–78^ in the case of the latter method. In the recently introduced adaptive CC­(*P*;*Q*) formalism,^126,127^ the triply excited determinants and amplitudes defining the leading *T*_3_ contributions in the iterative steps of CC­(*P*;*Q*) calculations are identified using the intrinsic structure of the CC­(*P*;*Q*) energy corrections.

In this study, we focus on the CIPSI-driven CC­(*P*;*Q*) methodology of ref 125. Our main goal is to answer the question of how efficient this methodology is in converging the lowest singlet and triplet potentials of cyclobutadiene along the *D*_2h_-symmetric automerization coordinate resulting from the high-level CCSDT computations shown in Figure 1. The ability of the CC­(*P*;*Q*) framework using the CIPSI algorithm to identify the leading triply excited determinants for the iterative steps of the CC­(*P*;*Q*) procedure to accurately approximate the CCSDT energies of the ^1^ *A*_*g*_(*D*_2h_) and ^3^ *B*_1*g*_(*D*_2h_) states of cyclobutadiene and the gap between them, especially at the most challenging TS geometry, in calculations using basis sets larger than cc-pVDZ (represented in this work by cc-pVTZ^83^) is examined as well. As shown in our initial study,^125^ the CIPSI-driven CC­(*P*;*Q*) approach is capable of producing the near-CCSDT energetics for singlet electronic states using tiny fractions of triply excited cluster amplitudes in the iterative parts of the CC­(*P*;*Q*) algorithm that are smaller than those used in the analogous semistochastic and active-orbital-based CC­(*P*;*Q*) considerations. One of the objectives of this work is to determine if similar observations apply to the CIPSI-driven CC­(*P*;*Q*) calculations for the lowest singlet and triplet potentials of cyclobutadiene along its automerization coordinate and the gap between them. The role of the CC­(*P*;*Q*) moment corrections in accelerating convergence toward CCSDT and the key elements of our improved implementation of the CIPSI-based CC­(*P*;*Q*) method, capable of efficiently handling small but generally spotty subsets of triply excited determinants in the underlying CC iterations, illustrated by computational timings, are discussed, too.

## 2 Theory and Computational Details

We begin by summarizing the key ingredients of the CC­(*P*;*Q*) formalism as applied to the ground-state problem or, in general, to the lowest state of a given symmetry for which a suitable single-determinantal reference can be found. Each CC­(*P*;*Q*) calculation requires defining two disjoint subspaces of the many-electron Hilbert space, called the *P* and *Q* spaces, designated as $H^{\left(P\right)}$ and $H^{\left(Q\right)}$ , respectively. The former space consists of the excited determinants |Φ_*K*_⟩ = *E*_*K*_|Φ⟩ which, together with the reference function |Φ⟩, dominate the electronic state of interest (*E*_*K*_ is the elementary particle–hole excitation operator that generates |Φ_*K*_⟩ from |Φ⟩). The determinants spanning the complementary *Q* space $H^{\left(Q\right)}$ are used to form the noniterative correction δ­(*P*;*Q*) which captures higher-order correlation effects the CC calculations in the *P* space do not describe.

All CC­(*P*;*Q*) computations consist of two stages. In the first, iterative, stage, denoted as CC­(*P*), we solve the CC amplitude equations in $H^{\left(P\right)}$ to determine amplitudes *t*_*K*_ that define the *P*-space cluster operator

3 $$T^{\left(P\right)}=\underset{\left|\Phi_{K}\right\rangle \in H^{\left(P\right)}}{\sum }t_{K}E_{K}.$$

This is done by employing the conventional projective technique adopted in the majority of single-reference CC calculations, i.e., by solving the system

4 $$M_{K}\left(P\right)=0,,\left|\Phi_{K}\right\rangle \in H^{\left(P\right)},$$

where

5 $$M_{K}\left(P\right)=\left\langle \Phi_{K}\right|{\mathrm{H̅}}^{\left(P\right)}|\Phi \rangle ,$$

with *H̅* ^(*P*)^ = *e* ^−*T* ^(*P*)^^ *H* *e* ^*T* ^(*P*)^^ representing the similarity-transformed Hamiltonian, are generalized moments of the CC­(*P*) equations.^96,97,146^ Once the cluster amplitudes *t*_*K*_ defining *T* ^(*P*)^ are determined, the CC­(*P*) energy is calculated in a usual way as

6 $$E^{\left(P\right)}=\left\langle \Phi \right|{\mathrm{H̅}}^{\left(P\right)}|\Phi \rangle .$$

In the second stage of the CC­(*P*;*Q*) procedure, we construct the aforementioned noniterative correction δ­(*P*;*Q*) using the expression

7 $$\delta \left(P;Q\right)=\underset{\left|\Phi_{K}\right\rangle \in H^{\left(Q\right)}}{\sum }l_{K}\left(P\right)M_{K}(P),$$

where coefficients $l_{K}\left(P\right)$ multiplying moments $M_{K}\left(P\right)$ are defined as

8 $$l_{K}\left(P\right)=\left\langle \Phi \right|\left(1+\Lambda^{\left(P\right)}\right){\mathrm{H̅}}^{\left(P\right)}\left|\Phi_{K}\right\rangle /D_{K}^{\left(P\right)},$$

with *D*_*K*_^(*P*)^ = *E* ^(*P*)^ − ⟨Φ_*K*_|*H̅* ^(*P*)^|Φ_*K*_⟩ designating the relevant Epstein–Nesbet-like denominators. The hole–particle deexcitation operator

9 $$\Lambda^{\left(P\right)}=\underset{\left|\Phi_{K}\right\rangle \in H^{\left(P\right)}}{\sum }\lambda_{K}{\left(E_{K}\right)}^{†}$$

in eq 8, which defines the bra state ⟨Ψ̃^(*P*)^| = ⟨Φ|(1 + Λ^(*P*)^) *e* ^−*T* ^(*P*)^^ matching the CC­(*P*) ket state |Ψ^(*P*)^⟩ = *e* ^*T* ^(*P*)^^|Φ⟩, is obtained by solving the linear system

10 $$\left\langle \Phi \right|\left(1+\Lambda^{\left(P\right)}\right){\mathrm{H̅}}^{\left(P\right)}\left|\Phi_{K}\right\rangle =E^{\left(P\right)}\lambda_{K},\left|\Phi_{K}\right\rangle \in H^{\left(P\right)}.$$

The final CC­(*P*;*Q*) energy is obtained using the formula

11 $$E^{\left(P+Q\right)}=E^{\left(P\right)}+\delta (P;Q).$$

One of the main advantages of the CC­(*P*;*Q*) methodology is its flexibility. In particular, we can make a wide variety of conventional as well as unconventional choices of the *P* and *Q* spaces, adjusting them to the nature of the electronic states of interest and adopting different numerical procedures in their construction. Conventional choices for the *P* and *Q* spaces, based on the many-body ranks of the determinants included in them, result in the left-eigenstate CR-CC methods, such as the CR-CC­(2,3) approach discussed in the Introduction, in which the former space consists of all singly and doubly excited determinants and the latter space is spanned by all triples. We can, however, also make unconventional choices, including those adopted in the CC­(t;3), CC­(t,q;3), CC­(t,q;3,4), CC­(q;4), etc. hierarchy^22,105,109,115,116,121,127^ and the semistochastic,^32,122–124^ adaptive,^126,127^ and CIPSI-driven^125^ CC(*P*;*Q*) methods, mentioned in the Introduction as well, in which the suitably chosen subsets of higher-than-doubly excited determinants are incorporated into the underlying *P* spaces, in addition to all singles and doubles, to relax the lower-rank *T*_1_ and *T*_2_ clusters in the presence of their higher-rank counterparts, such as the leading *T*_3_ contributions, which the CCSD­(T), CR-CC­(2,3), Λ-CCSD­(T), and similar approaches are not designed to do. Having some higher-than-doubly excited determinants in the *P* space provides us with a straightforward and computationally efficient mechanism to account for the coupling between the lower- and higher-order components of the cluster operator, which cannot be neglected when *T*_*n*_ contributions with *n* > 2, such as *T*_3_, become large and nonperturbative, as is the case when the automerization barrier region of the lowest-energy singlet potential of cyclobutadiene is examined. This, in turn, allows us to recover the full CCSDT, CCSDTQ, and similar energetics without running into the very expensive, often prohibitive, computational costs associated with the high-level CC methods of this type.

In the case of the CIPSI-driven CC­(*P*;*Q*) approach, introduced in ref 125 and investigated in this study, the desired subsets of higher-than-doubly excited determinants incorporated into the underlying *P* spaces are identified with the help of sequences of relatively inexpensive Hamiltonian diagonalizations in systematically grown, recursively defined, subspaces of the many-electron Hilbert space, denoted as $V_{\mathrm{int}}^{\left(k\right)}$ , where *k* = 0, 1, 2, ... enumerates the consecutive CIPSI iterations. In doing so, we follow the CIPSI algorithm, originally proposed in ref 76, further developed in refs 77 and 78 , and available in the Quantum Package 2.0 software.^78^ Given our interest in using CIPSI, which is one of the selected CI approaches^76,147–149^ (see refs 150–158 for other examples), within the single-reference CC­(*P*;*Q*) framework, the initial subspaces $V_{\mathrm{int}}^{\left(0\right)}$ adopted in our work are always spanned by the restricted Hartree–Fock (RHF) or restricted open-shell Hartree–Fock (ROHF) determinants. Once $V_{\mathrm{int}}^{\left(0\right)}$ is defined, each subsequent subspace $V_{\mathrm{int}}^{\left(k+1\right)}$ with *k* ≥ 0 is constructed by enlarging its $V_{\mathrm{int}}^{\left(k\right)}$ predecessor with the subset of the leading singly and doubly excited determinants generated out of it, identified with the help of the many-body perturbation theory (MBPT). Thus, if |Ψ_*k*_^(CIPSI)^⟩ = $\underset{\left|\Phi_{I}\right\rangle \in V_{\mathrm{int}}^{\left(k\right)}}{\sum }c_{I}\left|\Phi_{I}\right\rangle$ and *E*_var,*k*_ are the CI wave function and energy obtained in $V_{\mathrm{int}}^{\left(k\right)}$ , and if the space of all singles and doubles out of |Ψ_*k*_^(CIPSI)^⟩ is designated as $V_{\mathrm{ext}}^{\left(k\right)}$ , the subset of determinants |Φ_α_⟩ ∈ $V_{\mathrm{ext}}^{\left(k\right)}$ selected for inclusion in $V_{\mathrm{int}}^{\left(k+1\right)}$ consists of those that have the largest *e*_α,*k*_^(2)^ = |⟨Φ_α_|*H*|Ψ_*k*_^(CIPSI)^⟩|^2^/​(*E*_var,*k*_ − ⟨Φ_α_|*H*|Φ_α_⟩) contributions to the perturbative correction Δ*E*_*k*_^(2)^ = $\underset{\left|\Phi_{\alpha }\right\rangle \in V_{\mathrm{ext}}^{\left(k\right)}}{\sum }e_{\alpha ,k}^{\left(2\right)}$ to *E*_var,*k*_. Their selection is accomplished by arranging the sampled determinants |Φ_α_⟩ ∈ $V_{\mathrm{ext}}^{\left(k\right)}$ in descending order according to their |*e*_α,*k*_^(2)^| values and enlarging $V_{\mathrm{int}}^{\left(k\right)}$ , determinant by determinant, starting with the |Φ_α_⟩s associated with the largest |*e*_α,*k*_^(2)^| contributions and moving toward those characterized by smaller values of |*e*_α,*k*_^(2)^|, until the dimension of $V_{\mathrm{int}}^{\left(k+1\right)}$ exceeds that of its $V_{\mathrm{int}}^{\left(k\right)}$ predecessor by a user-defined factor *f* > 1, which in all the calculations performed in this study was set to its default value of 2 [the actual number of determinants included in $V_{\mathrm{int}}^{\left(k+1\right)}$ is usually slightly larger than *f* times the dimension of $V_{\mathrm{int}}^{\left(k\right)}$ since one may have to add extra determinants in $V_{\mathrm{int}}^{\left(k+1\right)}$ to make sure that the corresponding CI wave function |Ψ_*k*+1_^(CIPSI)^⟩ is an eigenstate of the total spin *S* ^2^ and *S*_*z*_ operators]. To reduce the computational costs associated with the above procedure of enlarging the $V_{\mathrm{int}}^{\left(k\right)}$ space to obtain $V_{\mathrm{int}}^{\left(k+1\right)}$ , in all the CIPSI-driven CC­(*P*;*Q*) computations reported in this work, we relied on a semistochastic version of the above determinant selection algorithm implemented in Quantum Package 2.0, in which one stochastically filters out the most important singly and doubly excited determinants out of |Ψ_*k*_^(CIPSI)^⟩, so that only a small subset of singles and doubles ends up in the $V_{\mathrm{ext}}^{\left(k\right)}$ space prior to determining and analyzing the *e*_α,*k*_^(2)^ contributions. The *e*_α,*k*_^(2)^ values, in addition to guiding the process of enlarging diagonalization spaces $V_{\mathrm{int}}^{\left(k\right)}$ and allowing us to evaluate the perturbatively corrected CIPSI energies *E*_var,*k*_ + Δ*E*_*k*_^(2)^, can be used to calculate the renormalized second-order corrections Δ*E*_r,*k*_^(2)^ introduced in ref 78 and the *E*_var,*k*_ + Δ*E*_r,*k*_^(2)^ energies.

To produce the final wave function |Ψ^(CIPSI)^⟩, needed to construct the list of higher-than-doubly excited determinants to be included in the *P* space of a given CIPSI-driven CC­(*P*;*Q*) calculation, and determine the associated variational (*E*_var_) and perturbatively corrected [*E*_var_ + Δ*E* ^(2)^ or *E*_var_ + Δ*E*_r_^(2)^] CIPSI energies, the sequence of Hamiltonian diagonalizations defining the underlying CIPSI run must be terminated. This could be done by stopping at the first iteration *k* for which the absolute value of the second-order MBPT correction Δ*E*_*k*_^(2)^ falls below a user-defined threshold η, but, given our interest in examining the convergence of the CIPSI-based CC­(*P*;*Q*) calculations toward the desired high-level CC energetics, represented in this study by CCSDT, using systematically grown *P* spaces obtained with the help of CIPSI, in this work we follow ref 125 and stop when the number of determinants in the diagonalization space equalizes or exceeds the user-defined parameter *N*_det(in)_. To ensure that the CIPSI sequences preceding our CC­(*P*;*Q*) calculations did not terminate too soon, before the dimensions of terminal diagonalization spaces became greater than or equal to *N*_det(in)_, we set the aforementioned parameter η to 1 microhartree. As a result [putting aside the convergence threshold used in the CC­(*P*) iterations, which we set to 10^–7^ hartree], all CIPSI-driven CC­(*P*;*Q*) computations reported in this article, along with the underlying *P* spaces, were controlled by a single input variable *N*_det(in)_. In addition to *N*_det(in)_, in presenting our CC­(*P*) and CC­(*P*;*Q*) results for the lowest singlet and triplet potentials of cyclobutadiene, we also provide information about the numbers of determinants included in the terminal CIPSI wave functions |Ψ^(CIPSI)^⟩ obtained for various values of *N*_det(in)_, designated as *N*_det(out)_. Given our choice of the subspace enlargement parameter *f*, the *N*_det(out)_ values characterizing the |Ψ^(CIPSI)^⟩ states used to identify the triply excited determinants for inclusion in the *P* spaces employed in our CIPSI-driven CC­(*P*;*Q*) computations were always between *N*_det(in)_ and 2*N*_det(in)_. With all of this in mind, the algorithm used in the CIPSI-enabled CC­(*P*;*Q*) calculations reported in this study, aimed at converging the CCSDT energetics, can be summarized as follows:^125^

(1) Choose a wave function termination parameter *N*_det(in)_ and execute a CIPSI diagonalization sequence starting from the one-dimensional subspace $V_{\mathrm{int}}^{\left(0\right)}$ spanned by the RHF or ROHF reference determinant |Φ⟩ to obtain the |Ψ^(CIPSI)^⟩ state.
(2) Extract the list of triply excited determinants included in |Ψ^(CIPSI)^⟩ and combine it with all singly and doubly excited determinants relative to |Φ⟩ to obtain the *P* space for CC­(*P*;*Q*) calculations.
(3) Solve the CC­(*P*) amplitude equations, eq 4, to determine the cluster operator *T* ^(*P*)^ = *T*_1_ + *T*_2_ + *T*_3_^(CIPSI)^, where *T*_3_^(CIPSI)^ is the three-body component of *T* ^(*P*)^ defined using the list of triply excited determinants extracted from |Ψ^(CIPSI)^⟩, and energy *E* ^(*P*)^, eq 6. Solve the left-eigenstate CC­(*P*) system given by eq 10 to obtain the companion hole–particle deexcitation operator Λ^(*P*)^ = Λ_1_ + Λ_2_ + Λ_3_^(CIPSI)^ in which the triples entering Λ_3_^(CIPSI)^ are the same as those included in *T*_3_^(CIPSI)^.
(4) Calculate the noniterative correction δ­(*P*;*Q*), eq 7, in which the *Q* space is defined as the remaining triply excited determinants absent in |Ψ^(CIPSI)^⟩, and add it to *E* ^(*P*)^ to obtain the CC­(*P*;*Q*) energy *E* ^(*P+Q*)^, eq 11.

The above steps 1–4 can be repeated by increasing *N*_det(in)_. The entire process can be stopped when the difference between consecutive *E* ^(*P+Q*)^ values falls below some small, user-specified, convergence threshold.

In order to perform the CIPSI-driven CC­(*P*) and CC­(*P*;*Q*) calculations for the singlet and triplet PECs of cyclobutadiene along the automerization coordinate investigated in this work and examine their convergence toward the parent CCSDT potentials, we used the computer programs described in ref 125 and the newer implementation of the same methods in our open-source CCpy package available on GitHub.^159^ The former codes take advantage of our highly efficient, automatically generated, Fortran CC routines that were previously exploited in implementing the active-orbital-based^22,105,109,115,116^ and semistochastic^122–124^ CC­(*P*;*Q*) approaches. The latter codes, available in CCpy, use a hybrid Python–Fortran programming approach. All of our CIPSI-driven CC­(*P*) and CC­(*P*;*Q*) computer programs targeting CCSDT and our group’s computer-generated CC codes used to produce the parent CCSDT data are interfaced with the RHF, ROHF, and integral transformation routines in GAMESS.^160–162^ As already alluded to above, the lists of triply excited determinants used to construct the *P* spaces for the CC­(*P*) and CC­(*P*;*Q*) calculations corresponding to the various choices of the input parameter *N*_det(in)_ were extracted from the terminal CIPSI wave functions |Ψ^(CIPSI)^⟩ obtained with Quantum Package 2.0, whereas the complementary *Q* spaces, needed to determine the δ­(*P*;*Q*) corrections, consisted of the remaining triples absent in the |Ψ^(CIPSI)^⟩ states. We also used Quantum Package 2.0 to obtain the *E*_var_, *E*_var_ + Δ*E* ^(2)^, and *E*_var_ + Δ*E*_r_^(2)^ energies associated with the CIPSI runs that provided the lists of triples for the CC­(*P*) iterations. The results of the DEA-EOMCC­(4p-2h)­{*N*_u_} calculations discussed in the Introduction were carried out with the highly efficient DEA-EOMCC routines developed in ref 29, which became part of the official GAMESS distribution in 2023.

To obtain the desired insights into the convergence of the singlet and triplet potentials of cyclobutadiene toward their CCSDT counterparts, we adopted the strategy used in ref 125. Thus, for each nuclear geometry along the automerization pathway considered in this work, we carried out a series of CIPSI-driven CC­(*P*) and CC­(*P*;*Q*) calculations using the *P* and *Q* spaces derived from the increasingly large CIPSI wave functions obtained by varying *N*_det(in)_ in an approximately semilogarithmic manner. We started with *N*_det(in)_ = 1, where the CIPSI wave functions |Ψ^(CIPSI)^⟩ are the single determinants defining the reference states |Φ⟩ used in our CC computations (RHF in the case of the singlet and ROHF in the triplet case) and the resulting CC­(*P*) and CC­(*P*;*Q*) energies become identical to those obtained with CCSD and CR-CC­(2,3), respectively, and went all the way to *N*_det(in)_ = 10,000,000, to reflect on the fact that as the input variable *N*_det(in)_ becomes increasingly large and the CIPSI wave functions capture more and more triply excited determinants, the CC­(*P*;*Q*) energies *E* ^(*P+Q*)^ approach their CCSDT parents (becoming identical to them when all triply excited determinants are captured by the |Ψ^(CIPSI)^⟩ states). The energies obtained in the CC­(*P*) computations approach their CCSDT counterparts as well, but, as discussed in the next section, their convergence toward CCSDT is much slower than that observed in the CC­(*P*;*Q*) runs.

The ability of the CIPSI-driven CC­(*P*;*Q*) methodology to generate the CCSDT-level energetics using tiny fractions of triply excited determinants in the underlying *P* spaces captured by the relatively small Hamiltonian diagonalizations, observed in the calculations for the lowest ^1^ *A*_*g*_(*D*_2h_) and ^3^ *B*_1*g*_(*D*_2h_) potentials of cyclobutadiene reported in this study, results in enormous savings in the computational effort compared to CCSDT. Indeed, as explained in ref 125, the CIPSI runs using smaller *N*_det(in)_ values are much faster than those needed to reach convergence, the CC­(*P*) calculations using tiny fractions of triples in the *P* space are one or more orders of magnitude faster than the corresponding CCSDT computations, and the effort involved in obtaining the noniterative δ­(*P*;*Q*) corrections is similar to the determination of the triples corrections of CR-CC­(2,3) or CCSD­(T). As pointed out in refs 123 and 124 , the key to obtaining the desired computational efficiency in the CC­(*P*;*Q*) calculations lies in the development of an algorithm capable of offering significant speedups compared to the parent CC approach, such as CCSDT, when the lists of higher-than-doubly excited determinants included in the CC­(*P*) iterations do not necessarily form continuous manifolds, as is the case when these lists are created by the sequences of Hamiltonian diagonalizations of CIPSI adopted in this study and ref 125, the previously employed CIQMC/CCMC propagations,^32,122–124^ or the moment expansions defining the δ­(*P*;*Q*) corrections.^126,127^ In all of these cases, conventional diagrammatic (or algebraic) techniques assuming continuous excitation manifolds labeled by occupied and unoccupied orbitals from the respective ranges of indices, used to implement the standard CC methods employing rank-based truncations, no longer apply. A different programming approach is needed. The key elements of our algorithm used to implement the CC­(*P*) equations in which the *P* space consists of all singly and doubly excited determinants and a generally spotty subset of triply excited determinants identified in this work by CIPSI, along with illustrative computational timings obtained using our CCpy codes,^159^ are summarized in the Appendix (for the analogous timings information obtained in the context of the adaptive CC­(*P*;*Q*) calculations for cyclobutadiene, see ref 126).

Two different basis sets were employed in this work, namely, cc-pVDZ, which we also used to construct the lowest-energy ^1^ *A*_*g*_(*D*_2h_) and ^3^ *B*_1*g*_(*D*_2h_) potentials of cyclobutadiene shown in Figure 1, and cc-pVTZ, used to discuss the effect of the basis set on our conclusions regarding the performance of the CIPSI-driven CC­(*P*;*Q*) approach. In generating the results of the CIPSI-based CC­(*P*;*Q*) computations for the lowest singlet and triplet states of cyclobutadiene along its automerization coordinate at different values of the wave function terminating parameter *N*_det(in)_ using a smaller cc-pVDZ basis, we adopted the same philosophy as that exploited in the case of the DEA-EOMCC­(4p-2h)­{*N*_u_}, extrapolated *E*_var_ + Δ*E*_r_^(2)^, and parent CCSDT potentials shown in Figure 1. Thus, we used eq 2, in which the geometries of the R and TS structures on the singlet potential obtained in the MR-AQCC/cc-pVDZ optimizations were taken from ref 40, to set up a one-dimensional, *D*_2h_-symmetric, automerization pathway parametrized by the dimensionless variable λ ∈ [0,1], and then, in analogy to the PECs shown in Figure 1, we executed our CIPSI-based CC­(*P*) and CC­(*P*;*Q*) calculations for the lowest ^1^ *A*_*g*_(*D*_2h_) and ^3^ *B*_1*g*_(*D*_2h_) states of cyclobutadiene and determined the corresponding *E*_var_, *E*_var_ + Δ*E* ^(2)^, and *E*_var_ + Δ*E*_r_^(2)^ CIPSI energies for λ = 0, 0.2, 0.4, 0.6, 0.8, and 1, reflecting the resulting PEC segments about λ = 1 to obtain the potentials that connect the rectangular reactant and product minima via the square TS. We used a similar strategy in the CIPSI-driven CC­(*P*) and CC­(*P*;*Q*) computations and the preceding CIPSI runs employing the cc-pVTZ basis set, but in this case, in reporting our results, we limited ourselves to the key R (λ = 0) and TS (λ = 1) structures optimized at the MR-AQCC/cc-pVTZ level in ref 40. Consistent with the overall symmetry of the automerization pathway examined in this work, the *D*_2h_ point group was adopted throughout. In particular, the *P* and *Q* spaces used in our CC­(*P*;*Q*) calculations for the lowest-energy singlet PEC consisted of the *S*_*z*_ = 0 determinants of the *A*_*g*_(*D*_2h_) symmetry. In the case of the lowest-energy triplet potential, they consisted of the *S*_*z*_ = 1 *B*_1*g*_(*D*_2h_) determinants. In all post-RHF/ROHF computations reported in this article, the four core molecular orbitals correlating with the 1s shells of the carbon atoms were frozen.

While the numerical evidence discussed in the next section clearly demonstrates that the CIPSI computations characterized by *N*_det(in)_ = 10,000,000 result in the *P* spaces that are unnecessarily large for accurately approximating the CCSDT energetics using the CIPSI-driven CC­(*P*;*Q*) approach when the cc-pVDZ and cc-pVTZ basis sets are employed, we include them in our analysis since they also allowed us to extrapolate the near-full-CI *E*_var_ + Δ*E*_r_^(2)^ potentials, such as those presented in Figure 1 for a cc-pVDZ basis, by following the procedure described in refs 41, 78, and 163 (see, also, ref 125). In this procedure, the *E*_var,*k*_ + Δ*E*_r,*k*_^(2)^ energies extracted from the last four to six Hamiltonian diagonalizations of the CIPSI sequence leading to the final |Ψ^(CIPSI)^⟩ state are plotted against the corresponding Δ*E*_r,*k*_^(2)^ corrections and the resulting data, fit to a line, are extrapolated to the Δ*E*_r_^(2)^ = 0 limit. In the case of the CIPSI calculations employing the cc-pVDZ basis set, to extrapolate the reasonably smooth PECs for the lowest singlet and triplet states of cyclobutadiene out of our largest CIPSI runs corresponding to *N*_det(in)_ = 10,000,000, shown in Figure 1, we used the last six *E*_var,*k*_ + Δ*E*_r,*k*_^(2)^ and Δ*E*_r,*k*_^(2)^ values of each of these runs, since using fewer than six values resulted in unphysical bumps in the extrapolated potentials. The determination of the extrapolated *E*_var_ + Δ*E*_r_^(2)^ energies of the lowest ^1^ *A*_*g*_(*D*_2h_) and ^3^ *B*_1*g*_(*D*_2h_) states of cyclobutadiene at the R and TS geometries using the cc-pVTZ basis had to be handled differently. In this case, we relied on the last four *E*_var,*k*_ + Δ*E*_r,*k*_^(2)^ and Δ*E*_r,*k*_^(2)^ values obtained in the *N*_det(in)_ = 10,000,000 CIPSI runs. Using more values than four led to problems with producing a sensible result for the extrapolated *E*_var_ + Δ*E*_r_^(2)^ energy of the lowest singlet state at the TS geometry. In addition to allowing us to comment on the quality of the CCSDT energetics in the Introduction, the extrapolated *E*_var_ + Δ*E*_r_^(2)^ energies serve in this study as the reference data for assessing the accuracy of the *E*_var_, *E*_var_ + Δ*E* ^(2)^, and *E*_var_ + Δ*E*_r_^(2)^ values obtained in the CIPSI calculations using various choices of *N*_det(in)_ from a 1–10,000,000 range.

## 3 Results and Discussion

As explained in the Introduction, the primary objective of this study is to examine the efficiency of the CIPSI-driven CC­(*P*;*Q*) methodology in converging the full CCSDT data for the lowest singlet and triplet potentials of cyclobutadiene along its automerization coordinate and the gap between them. We are especially interested in investigating how effective the CIPSI-driven CC­(*P*;*Q*) approach is in balancing the substantial nondynamical many-electron correlation effects characterizing the lowest ^1^ *A*_*g*_(*D*_2h_) state in the neighborhood of the automerization barrier region, where *T*_3_ clusters become large, nonperturbative, and strongly coupled to their lower-rank *T*_1_ and *T*_2_ counterparts, with the predominantly dynamical correlations characterizing the lowest ^3^ *B*_1*g*_(*D*_2h_) state. As pointed out in the Introduction, using comparisons with the DEA-EOMCC­(4p-2h)­{*N*_u_} and perturbatively corrected and extrapolated CIPSI results, the CCSDT approach, despite its intrinsically single-reference character, captures essentially all relevant many-electron correlation effects needed to accurately describe the lowest singlet and triplet potentials of cyclobutadiene and the separation between them. The question is if the CIPSI-driven CC­(*P*;*Q*) computations using compact wave functions |Ψ^(CIPSI)^⟩, resulting from the relatively inexpensive CIPSI diagonalization sequences characterized by the *N*_det(out)_ values that are much smaller than the numbers of all *T*_3_ amplitudes, and tiny fractions of the triply excited determinants in the underlying *P* spaces are capable of accomplishing the same. We also examine how effective the noniterative δ­(*P*;*Q*) corrections are in accelerating convergence of the CC­(*P*) energetics toward their CCSDT parents and how the rate of convergence of the CIPSI-driven CC­(*P*;*Q*) calculations toward CCSDT with *N*_det(in)_ compares to the analogous convergence of the perturbatively corrected CIPSI energies toward their extrapolated *E*_var_ + Δ*E*_r_^(2)^ values. Comparisons with the CCSDt and CC­(t;3) methods, which belong to the active-orbital-based CC­(*P*) and CC­(*P*;*Q*) hierarchies, and the effect of the basis set on the performance of the CIPSI-based CC­(*P*;*Q*) methodology in calculations of the lowest singlet and triplet states of cyclobutadiene are discussed as well.

We begin by analyzing our CIPSI-driven CC­(*P*) and CC­(*P*;*Q*) computations employing the cc-pVDZ basis set used in most of the calculations reported in this work. The results of our CIPSI-driven CC­(*P*)/cc-pVDZ and CC­(*P*;*Q*)/cc-pVDZ calculations for the lowest-energy ^1^ *A*_*g*_(*D*_2h_) and ^3^ *B*_1*g*_(*D*_2h_) potentials of cyclobutadiene along its automerization coordinate, the separation between them, and the associated *E*_var_, *E*_var_ + Δ*E* ^(2)^, and *E*_var_ + Δ*E*_r_^(2)^ data can be found in Tables 1–3. The convergence of the CC­(*P*)/cc-pVDZ and CC­(*P*;*Q*)/cc-pVDZ energies of the lowest singlet and triplet states of cyclobutadiene and the gaps between them, determined at λ = 0, 0.2, 0.4, 0.6, 0.8, and 1, toward their CCSDT/cc-pVDZ parents as functions of the parameters *N*_det(in)_ and *N*_det(out)_ that control [*N*_det(in)_] and define [*N*_det(out)_] the sizes of the terminal wave functions |Ψ^(CIPSI)^⟩ produced by the underlying CIPSI runs is also visualized in Figures 2–4 and, in a summary form, in Figure 5.

**Table 1:** Convergence of the CC­(*P*) and CC­(*P*;*Q*) Energies of the Lowest Singlet State of Cyclobutadiene, as Described by the cc-pVDZ Basis Set, Toward CCSDT at Selected Values of Parameter λ Defining the Automerization Coordinate via the Interpolation Formula Given by Eq 2, Alongside the Associated Variational and Perturbatively Corrected CIPSI Energies

λ	*N*_det(in)_/*N*_det(out)_	% of triples	*E*_var_ ^a^	*E*_var_ + Δ*E* ^(2)^ ^a^	*E*_var_ + Δ*E*_r_^(2)^ ^a^	CC(*P*)^b^	CC(*P*;*Q*)^b^
0	1/1	0	596.966^c^	–84.890^d^	119.654	26.827^e^	0.848^f^
	50,000/55,651	0.0	120.631	25.127(179)	27.145(175)	25.481	0.676
	100,000/111,316	0.1	107.950	22.116(146)	23.686(143)	22.183	0.431
	250,000/445,296	0.6	95.932	18.756(144)	19.974(141)	17.706	0.278
	500,000/890,920	1.1	91.033	17.669(142)	18.752(140)	16.258	0.268
	1,000,000/1,781,339	2.2	86.494	16.576(139)	17.543(137)	14.608	0.254
	5,000,000/7,127,768	7.7	75.839	14.895(121)	15.604(119)	10.733	0.145
	10,000,000/14,258,080	15.1	67.232	13.219(108)	13.759(107)	7.252	0.093
0.2	1/1	0	601.559^c^	–87.141^d^	129.221	27.964^e^	1.253^f^
	50,000/51,630	0.0	126.605	27.112(167)	29.321(164)	26.641	1.012
	100,000/103,165	0.1	111.522	24.556(150)	26.178(147)	23.029	0.552
	250,000/412,603	0.5	98.105	19.604(152)	20.870(149)	17.911	0.272
	500,000/825,242	1.1	92.595	18.791(145)	19.889(143)	16.130	0.270
	1,000,000/1,651,057	2.0	87.874	17.728(135)	18.703(133)	14.653	0.264
	5,000,000/6,602,235	7.3	77.378	15.889(122)	16.612(121)	10.783	0.148
	10,000,000/13,223,732	13.9	68.937	14.333(109)	14.887(107)	7.524	0.101
0.4	1/1	0	605.168^c^	–91.486^d^	141.934	29.667^e^	2.021^f^
	50,000/50,677	0.0	129.124	28.480(178)	30.760(174)	28.021	1.563
	100,000/101,361	0.1	113.415	24.995(166)	26.686(163)	23.947	0.776
	250,000/405,591	0.5	98.290	19.122(156)	20.416(153)	18.165	0.271
	500,000/811,227	1.1	92.053	17.563(147)	18.682(145)	15.984	0.292
	1,000,000/1,621,981	2.0	87.558	17.149(139)	18.133(137)	14.611	0.274
	5,000,000/6,488,516	7.2	76.901	15.082(123)	15.813(122)	10.710	0.160
	10,000,000/12,976,521	10.7	73.466	14.493(118)	15.156(116)	10.238	0.115
0.6	1/1	0	610.659^c^	–95.640^d^	164.690	32.473^e^	3.582^f^
	50,000/53,206	0.0	130.859	30.784(188)	33.041(184)	30.198	2.679
	100,000/106,413	0.1	115.914	26.643(175)	28.371(171)	25.279	1.235
	250,000/425,835	0.5	98.110	18.561(152)	19.866(150)	17.603	0.300
	500,000/851,740	1.1	91.503	17.269(143)	18.381(140)	15.781	0.304
	1,000,000/1,703,867	2.0	87.073	16.711(139)	17.693(137)	14.282	0.284
	5,000,000/6,812,598	7.5	75.664	14.470(122)	15.183(121)	9.967	0.167
	10,000,000/13,627,034	13.9	68.381	13.315(108)	13.878(107)	7.311	0.109
0.8	1/1	0	619.744^c^	–98.958^d^	207.413	37.662^e^	7.008^f^
	50,000/98,465	0.1	123.879	32.073(170)	33.919(167)	28.703	2.505
	100,000/196,965	0.2	112.359	24.572(171)	26.222(168)	21.842	0.628
	250,000/394,080	0.5	100.961	19.800(151)	21.171(148)	17.926	0.336
	500,000/787,924	1.0	93.638	18.350(149)	19.500(147)	15.451	0.360
	1,000,000/1,575,423	1.9	88.146	17.491(137)	18.484(135)	13.731	0.327
	5,000,000/6,300,768	6.0	78.214	15.623(125)	16.375(123)	10.615	0.211
	10,000,000/12,604,257	10.7	71.699	14.195(115)	14.816(113)	8.367	0.150
1	1/1	0	632.766^c^	–102.757^d^	282.305	47.979^e^	14.636^f^
	50,000/56,219	0.0	146.883	45.172(210)	47.519(205)	42.119	9.569
	100,000/112,432	0.1	130.721	36.708(182)	38.658(178)	32.125	3.539
	250,000/449,753	0.5	99.218	19.367(152)	20.688(150)	17.137	0.458
	500,000/899,464	0.9	92.482	17.938(148)	19.059(145)	14.685	0.435
	1,000,000/1,799,702	1.7	87.614	17.261(140)	18.243(138)	13.223	0.375
	5,000,000/7,196,961	5.5	77.242	15.348(125)	16.078(123)	9.969	0.246
	10,000,000/14,391,011	9.6	71.571	14.183(114)	14.800(113)	8.486	0.167

a For each value of λ, the *E*_var_, *E*_var_ + Δ*E* ^(2)^, and *E*_var_ + Δ*E*_r_^(2)^ energies are reported as errors, in millihartree, relative to the extrapolated *E*_var_ + Δ*E*_r_^(2)^ energy found using a linear fit based on the last six *E*_var,*k*_ + Δ*E*_r,*k*_^(2)^ values leading to the largest CIPSI wave function obtained with *N*_det(in)_ = 10,000,000, plotted against the corresponding Δ*E*_r,*k*_^(2)^ corrections, following the procedure described in refs 41, 78, and 163 . The extrapolated *E*_var_ + Δ*E*_r_^(2)^ energies at λ = 0, 0.2, 0.4, 0.6, 0.8, and 1 are −154.248137(398), −154.247883(480), −154.244213(872), −154.239997(642), −154.236928(616), and −154.235401(1043) hartree, respectively, where the error bounds in parentheses correspond to the uncertainty associated with the linear fit. The error bounds for the *E*_var_ + Δ*E* ^(2)^ and *E*_var_ + Δ*E*_r_^(2)^ energies obtained at the various values of *N*_det(in)_ reflect on the semistochastic design of the $V_{\mathrm{ext}}^{\left(k\right)}$ spaces discussed in the main text, but they ignore the uncertainties characterizing the reference *E*_var_ + Δ*E*_r_^(2)^ energies obtained in the above extrapolation procedure.

b The CC­(*P*) and CC­(*P*;*Q*) energies are reported as errors relative to CCSDT, in millihartree. The total CCSDT energies at λ = 0, 0.2, 0.4, 0.6, 0.8, and 1 are −154.244157, −154.242922, −154.240027, −154.236079, −154.232439, and −154.232002 hartree, respectively.

c Equivalent to RHF.

d Equivalent to the result obtained with the second-order MBPT approach using the Epstein–Nesbet denominator.

e Equivalent to CCSD.

f Equivalent to CR-CC­(2,3).

**Table 2:** Convergence of the CC­(*P*) and CC­(*P*;*Q*) Energies of the Lowest Triplet State of Cyclobutadiene, as Described by the cc-pVDZ Basis Set, Toward CCSDT at Selected Values of Parameter λ Defining the Automerization Coordinate via the Interpolation Formula Given by Eq 2, Alongside the Associated Variational and Perturbatively Corrected CIPSI Energies

λ	*N*_det(in)_/*N*_det(out)_	% of triples	*E*_var_ ^a^	*E*_var_ + Δ*E* ^(2)^ ^a^	*E*_var_ + Δ*E*_r_^(2)^ ^a^	CC(*P*)^b^	CC(*P*;*Q*)^b^
0	1/1	0	572.232^c^	–97.167^d^	94.195	24.646^e^	–0.033^f^
	50,000/84,925	0.3	126.216	17.881(210)	20.609(205)	22.589	0.016
	100,000/169,861	0.5	100.010	14.692(155)	16.283(152)	20.446	0.028
	250,000/339,721	0.8	90.992	13.111(144)	14.389(142)	18.341	0.100
	500,000/679,710	1.2	85.449	12.584(139)	13.672(137)	16.737	0.154
	1,000,000/1,359,265	1.8	81.995	12.345(137)	13.324(135)	15.567	0.173
	5,000,000/5,436,202	4.6	74.316	11.275(126)	12.053(124)	12.474	0.167
	10,000,000/10,871,115	7.7	69.341	10.222(118)	10.896(117)	10.637	0.134
0.2	1/1	0	571.775^c^	–95.504^d^	93.218	24.424^e^	–0.043^f^
	50,000/95,659	0.3	119.164	17.033(194)	19.415(189)	22.170	0.015
	100,000/191,346	0.6	97.554	14.541(153)	16.027(151)	19.833	0.072
	250,000/382,772	0.8	90.002	12.999(155)	14.237(152)	17.991	0.100
	500,000/765,329	1.3	84.848	12.854(143)	13.912(141)	16.326	0.155
	1,000,000/1,532,203	2.0	80.932	12.414(135)	13.356(133)	14.846	0.173
	5,000,000/6,122,654	5.1	72.919	11.032(121)	11.776(120)	11.898	0.152
	10,000,000/12,246,843	8.5	67.669	10.147(114)	10.778(113)	9.840	0.126
0.4	1/1	0	572.339^c^	–93.175^d^	93.387	24.239^e^	–0.050^f^
	50,000/68,315	0.2	138.142	20.184(191)	23.481(186)	22.835	–0.028
	100,000/136,635	0.4	105.157	16.968(158)	18.675(155)	20.901	0.003
	250,000/273,285	0.7	94.039	14.997(147)	16.319(145)	18.707	0.068
	500,000/546,881	1.0	88.089	14.172(147)	15.295(144)	17.028	0.130
	1,000,000/1,093,480	1.6	84.427	13.398(141)	14.420(139)	15.794	0.154
	5,000,000/8,746,894	6.6	71.913	12.125(119)	12.815(118)	10.994	0.138
	10,000,000/17,483,610	12.1	62.833	10.294(105)	10.813(104)	8.130	0.084
0.6	1/1	0	570.893^c^	–93.217^d^	91.663	24.089^e^	–0.055^f^
	50,000/55,070	0.2	150.423	19.777(414)	23.900(401)	23.037	–0.034
	100,000/110,142	0.4	110.889	18.391(125)	20.292(122)	21.542	–0.015
	250,000/440,697	0.9	88.802	13.367(142)	14.547(140)	17.540	0.096
	500,000/881,321	1.3	84.455	13.032(131)	14.069(129)	16.110	0.143
	1,000,000/1,762,363	2.1	80.638	12.620(131)	13.546(130)	14.705	0.162
	5,000,000/7,051,421	5.5	73.209	11.431(124)	12.173(122)	11.869	0.132
	10,000,000/14,099,214	9.1	67.881	10.554(114)	11.183(113)	9.951	0.117
0.8	1/1	0	570.863^c^	–92.204^d^	91.469	23.974^e^	–0.058^f^
	50,000/59,298	0.2	144.443	20.389(241)	24.065(234)	22.846	–0.040
	100,000/118,602	0.4	107.656	17.516(184)	19.303(180)	21.062	–0.006
	250,000/474,464	0.9	88.836	14.143(145)	15.297(143)	17.489	0.094
	500,000/949,394	1.4	85.025	13.589(140)	14.628(138)	16.201	0.131
	1,000,000/1,898,021	2.2	81.984	13.095(133)	14.049(131)	15.224	0.131
	5,000,000/7,591,707	5.5	73.852	11.863(123)	12.613(122)	12.110	0.125
	10,000,000/15,188,890	9.1	68.485	11.085(113)	11.716(112)	10.195	0.099
1	1/1	0	570.406^c^	–91.860^d^	90.994	23.884^e^	–0.060^f^
	50,000/65,391	0.2	137.892	20.022(218)	23.305(212)	22.617	–0.047
	100,000/130,810	0.4	103.950	16.288(153)	17.965(150)	20.624	–0.010
	250,000/261,626	0.6	93.518	14.821(148)	16.127(145)	18.665	0.039
	500,000/523,285	0.9	87.775	13.661(137)	14.792(134)	17.237	0.109
	1,000,000/1,046,443	1.4	84.673	13.507(140)	14.536(137)	16.189	0.127
	5,000,000/8,373,419	5.8	74.128	11.775(124)	12.535(122)	12.371	0.117
	10,000,000/16,741,696	9.3	68.611	11.074(115)	11.711(114)	10.435	0.101

a For each value of λ, the *E*_var_, *E*_var_ + Δ*E* ^(2)^, and *E*_var_ + Δ*E*_r_^(2)^ energies are reported as errors, in millihartree, relative to the extrapolated *E*_var_ + Δ*E*_r_^(2)^ energy found using a linear fit based on the last six *E*_var.*k*_ + Δ*E*_r,*k*_^(2)^ values leading to the largest CIPSI wave function obtained with *N*_det(in)_ = 10,000,000, plotted against the corresponding Δ*E*_r,*k*_^(2)^ corrections, following the procedure described in refs 41, 78, and 163 . The extrapolated *E*_var_ + Δ*E*_r_^(2)^ energies at λ = 0, 0.2, 0.4, 0.6, 0.8, and 1 are −154.195674(1195), −154.206430(1047), −154.215793(862), −154.220754(582), −154.224733(1124), and −154.225942(668) hartree, respectively, where the error bounds in parentheses correspond to the uncertainty associated with the linear fit. The error bounds for the *E*_var_ + Δ*E* ^(2)^ and *E*_var_ + Δ*E*_r_^(2)^ energies obtained at the various values of *N*_det(in)_ reflect on the semistochastic design of the $V_{\mathrm{ext}}^{\left(k\right)}$ spaces discussed in the main text, but they ignore the uncertainties characterizing the reference *E*_var_ + Δ*E*_r_^(2)^ energies obtained in the above extrapolation procedure.

b The CC­(*P*) and CC­(*P*;*Q*) energies are reported as errors relative to CCSDT, in millihartree. The total CCSDT energies at λ = 0, 0.2, 0.4, 0.6, 0.8, and 1 are −154.195389, −154.205779, −154.213867, −154.219672, −154.223190, and −154.224380 hartree, respectively.

c Equivalent to ROHF.

d Equivalent to the result obtained with the second-order MBPT approach using the Epstein–Nesbet denominator.

e Equivalent to CCSD.

f Equivalent to CR-CC­(2,3).

**Table 3:** Convergence of the CC­(*P*) and CC­(*P*;*Q*) Singlet–Triplet Gaps Δ*E*_S–T_ = *E*_S_ – *E*_T_ Characterizing Cyclobutadiene, as Described by the cc-pVDZ Basis Set, Toward Their CCSDT Parents at Selected Values of Parameter λ Defining the Automerization Coordinate via the Interpolation Formula Given by Eq 2, Along with the Δ*E*_S–T_ Data Resulting from the Associated Variational and Perturbatively Corrected CIPSI Computations

λ	*N*_det(in)_/*N*_det(out)_	% of triples	*E*_var_ ^a^	*E*_var_ + Δ*E* ^(2)^ ^a^	*E*_var_ + Δ*E*_r_^(2)^ ^a^	CC(*P*)^b^	CC(*P*;*Q*)^b^
0	1/1; 1	0; 0	15.521^c^	7.704^d^	15.976	1.368^e^	0.553^f^
	50,000/55,651; 84,925	0.0; 0.3	–3.504	4.546(173)	4.101(169)	1.815	0.414
	100,000/111,316; 169,861	0.1; 0.5	4.982	4.658(133)	4.645(131)	1.090	0.253
	250,000/445,296; 339,721	0.6; 0.8	3.100	3.542(128)	3.505(126)	–0.398	0.112
	500,000/890,920; 679,710	1.1; 1.2	3.504	3.191(125)	3.188(123)	–0.300	0.071
	1,000,000/1,781,339; 1,359,265	2.2; 1.8	2.823	2.655(122)	2.647(121)	–0.602	0.051
	5,000,000/7,127,768; 5,436,202	7.7; 4.6	0.956	2.272(110)	2.228(108)	–1.092	–0.014
	10,000,000/14,258,080; 10,871,115	15.1; 7.7	–1.324	1.881(100)	1.797(099)	–2.124	–0.025
0.2	1/1; 1	0; 0	18.690^c^	5.248^d^	22.592	2.221^e^	0.813^f^
	50,000/51,630; 95,659	0.0; 0.3	4.670	6.325(161)	6.216(157)	2.805	0.626
	100,000/103,165; 191,346	0.1; 0.6	8.765	6.284(135)	6.370(132)	2.006	0.301
	250,000/412,603; 382,772	0.5; 0.8	5.085	4.145(136)	4.162(134)	–0.050	0.108
	500,000/825,242; 765,329	1.1; 1.3	4.861	3.726(128)	3.751(126)	–0.123	0.072
	1,000,000/1,651,057; 1,532,203	2.0; 2.0	4.357	3.334(120)	3.355(118)	–0.121	0.057
	5,000,000/6,602,235; 6,122,654	7.3; 5.1	2.798	3.048(108)	3.035(107)	–0.700	–0.003
	10,000,000/13,223,732; 12,246,843	13.9; 8.5	0.796	2.627(099)	2.578(098)	–1.453	–0.015
0.4	1/1; 1	0; 0	20.600^c^	1.060^d^	30.464	3.406^e^	1.300^f^
	50,000/50,677; 68,315	0.0; 0.2	–5.659	5.205(164)	4.567(160)	3.254	0.998
	100,000/101,361; 136,635	0.1; 0.4	5.182	5.037(144)	5.027(141)	1.911	0.485
	250,000/405,591; 273,285	0.5; 0.7	2.667	2.589(135)	2.570(132)	–0.340	0.128
	500,000/811,227; 546,881	1.1; 1.0	2.488	2.128(130)	2.125(128)	–0.655	0.102
	1,000,000/1,621,981; 1,093,480	2.0; 1.6	1.965	2.354(125)	2.330(123)	–0.743	0.076
	5,000,000/6,488,516; 8,746,894	7.2; 6.6	3.130	1.855(108)	1.881(106)	–0.178	0.014
	10,000,000/12,976,521; 17,483,610	10.7; 12.1	6.672	2.635(099)	2.725(098)	1.323	0.020
0.6	1/1; 1	0; 0	24.953^c^	–1.520^d^	45.826	5.261^e^	2.282^f^
	50,000/53,206; 55,070	0.0; 0.2	–12.277	6.907(285)	5.736(277)	4.493	1.703
	100,000/106,413; 110,142	0.1; 0.4	3.154	5.179(135)	5.070(132)	2.345	0.784
	250,000/425,835; 440,697	0.5; 0.9	5.841	3.259(131)	3.338(128)	0.040	0.128
	500,000/851,740; 881,321	1.1; 1.3	4.423	2.659(121)	2.706(120)	–0.207	0.101
	1,000,000/1,703,867; 1,762,363	2.0; 2.1	4.038	2.567(120)	2.602(119)	–0.266	0.077
	5,000,000/6,812,598; 7,051,421	7.5; 5.5	1.541	1.907(109)	1.889(108)	–1.193	0.022
	10,000,000/13,627,034; 14,099,214	13.9; 9.1	0.314	1.732(099)	1.691(098)	–1.657	–0.005
0.8	1/1; 1	0; 0	30.673^c^	–4.238^d^	72.756	8.589^e^	4.434^f^
	50,000/98,465; 59,298	0.1; 0.2	–12.905	7.332(151)	6.184(180)	3.676	1.597
	100,000/196,965; 118,602	0.2; 0.4	2.951	4.428(152)	4.342(154)	0.490	0.398
	250,000/394,080; 474,464	0.5; 0.9	7.609	3.550(134)	3.685(129)	0.274	0.152
	500,000/787,924; 949,394	1.0; 1.4	5.404	2.987(132)	3.057(126)	–0.471	0.143
	1,000,000/1,575,423; 1,898,021	1.9; 2.2	3.867	2.759(121)	2.783(118)	–0.937	0.123
	5,000,000/6,300,768; 7,591,707	6.0; 5.5	2.737	2.359(111)	2.360(109)	–0.938	0.054
	10,000,000/12,604,257; 15,188,890	10.7; 9.1	2.016	1.952(102)	1.945(100)	–1.147	0.032
1	1/1; 1	0; 0	39.131^c^	–6.838^d^	120.049	15.120^e^	9.222^f^
	50,000/56,219; 65,391	0.0; 0.2	5.642	15.782(190)	15.195(185)	12.238	6.035
	100,000/112,432; 130,810	0.1; 0.4	16.799	12.814(149)	12.985(146)	7.217	2.227
	250,000/449,753; 261,626	0.5; 0.6	3.577	2.853(133)	2.862(131)	–0.959	0.263
	500,000/899,464; 523,285	0.9; 0.9	2.954	2.683(126)	2.678(124)	–1.601	0.205
	1,000,000/1,799,702; 1,046,443	1.7; 1.4	1.845	2.356(124)	2.326(122)	–1.861	0.156
	5,000,000/7,196,961; 8,373,419	5.5; 5.8	1.954	2.243(110)	2.223(109)	–1.507	0.081
	10,000,000/14,391,011; 16,741,696	9.6; 9.3	1.857	1.951(101)	1.938(100)	–1.223	0.042


a For each value of λ, the *E*_var_, *E*_var_ + Δ*E* ^(2)^, and *E*_var_ + Δ*E*_r_^(2)^ singlet–triplet gaps are reported as errors, in kcal/mol, relative to the parent CIPSI data obtained by forming the differences between the extrapolated *E*_var_ + Δ*E*_r_^(2)^ energies of the lowest singlet and triplet states given in footnotes “a” of Tables 1 and 2. The resulting reference *E*_var_ + Δ*E*_r_^(2)^ singlet–triplet gap values at λ = 0, 0.2, 0.4, 0.6, 0.8, and 1 are −32.921(790), −26.013(723), −17.833(769), −12.076(544), −7.653(804), and −5.936(777) kcal/mol, respectively.

b The CC­(*P*) and CC­(*P*;*Q*) singlet–triplet gaps are reported as errors relative to CCSDT, in kcal/mol. The CCSDT singlet–triplet gap values at λ = 0, 0.2, 0.4, 0.6, 0.8, and 1 are −30.603, −23.308, −16.416, −10.295, −5.804, and −4.783 kcal/mol, respectively.

c Equivalent to RHF/ROHF.

d Equivalent to the result obtained with the second-order MBPT approach using the Epstein–Nesbet denominator.

e Equivalent to CCSD.

f Equivalent to CR-CC­(2,3).

**Figure 2:**
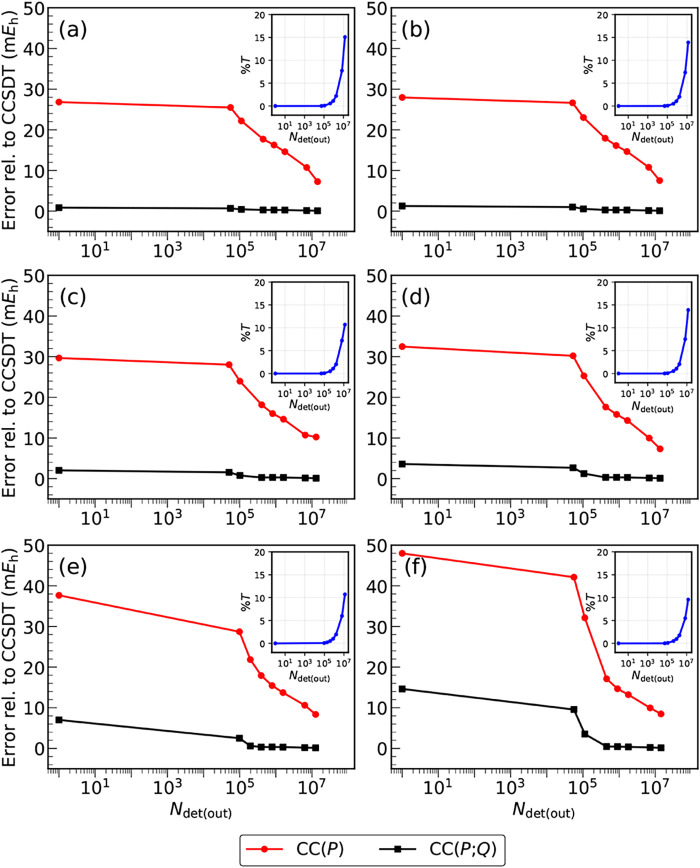
Graphical illustration of the convergence of the CC­(*P*) (red lines and circles) and CC­(*P*;*Q*) (black lines and squares) energies characterizing the lowest singlet state of cyclobutadiene, as described by the cc-pVDZ basis set, toward their CCSDT parents as functions of the actual numbers of determinants *N*_det(out)_ that define the sizes of the terminal wave functions |Ψ^(CIPSI)^⟩ generated in the underlying CIPSI runs at (a) λ = 0, (b) λ = 0.2, (c) λ = 0.4, (d) λ = 0.6, (e) λ = 0.8, and (f) λ = 1. The insets show the percentages of the *S*_*z*_ = 0 *A*_*g*_(*D*_2h_)-symmetric triply excited determinants captured by CIPSI as functions of *N*_det(out)_.

**Figure 3:**
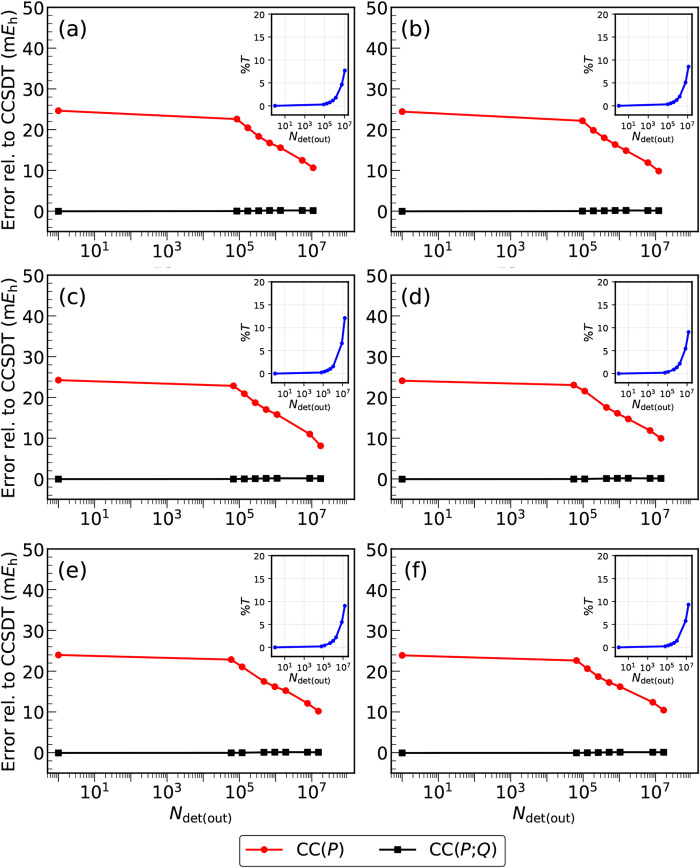
Graphical illustration of the convergence of the CC­(*P*) (red lines and circles) and CC­(*P*;*Q*) (black lines and squares) energies characterizing the lowest triplet state of cyclobutadiene, as described by the cc-pVDZ basis set, toward their CCSDT parents as functions of the actual numbers of determinants *N*_det(out)_ that define the sizes of the terminal wave functions |Ψ^(CIPSI)^⟩ generated in the underlying CIPSI runs at (a) λ = 0, (b) λ = 0.2, (c) λ = 0.4, (d) λ = 0.6, (e) λ = 0.8, and (f) λ = 1. The insets show the percentages of the *S*_*z*_ = 1 *B*_1*g*_(*D*_2h_)-symmetric triply excited determinants captured by CIPSI as functions of *N*_det(out)_.

**Figure 4:**
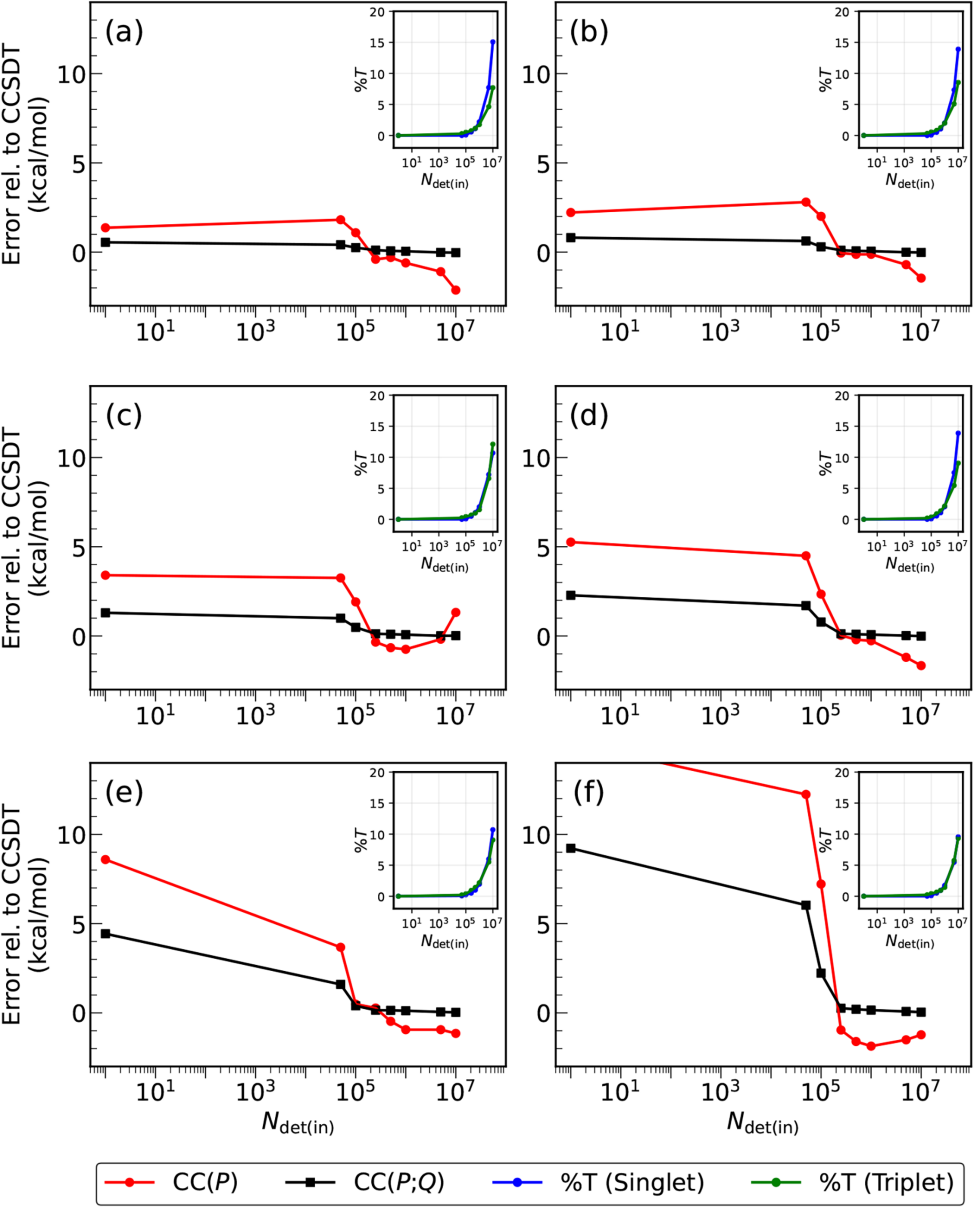
Graphical illustration of the convergence of the CC­(*P*) (red lines and circles) and CC­(*P*;*Q*) (black lines and squares) singlet–triplet gaps Δ*E*_S–T_ = *E*_S_ – *E*_T_ of cyclobutadiene, as described by the cc-pVDZ basis set, toward their CCSDT parents as functions of the CIPSI input parameter *N*_det(in)_ (common to the calculations for the lowest singlet and triplet states) at (a) λ = 0, (b) λ = 0.2, (c) λ = 0.4, (d) λ = 0.6, (e) λ = 0.8, and (f) λ = 1. The insets show the percentages of the triply excited determinants of the *S*_*z*_ = 0 *A*_*g*_(*D*_2h_) (blue lines and circles) and *S*_*z*_ = 1 *B*_1*g*_(*D*_2h_) (green lines and circles) symmetries captured by the underlying CIPSI runs as functions of *N*_det(in)_.

**Figure 5:**
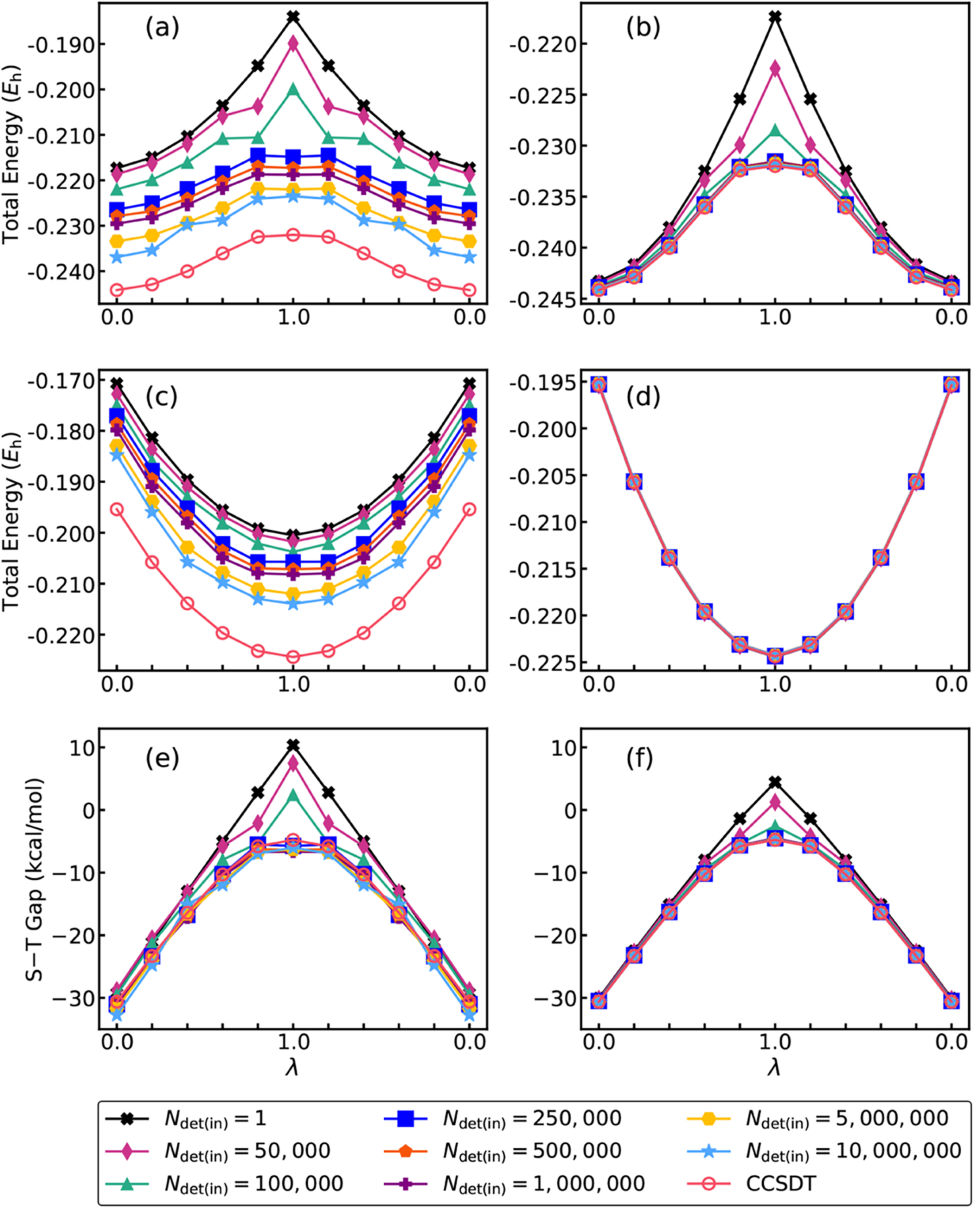
Convergence of the CC­(*P*) and CC­(*P*;*Q*) energies *E*, reported as (*E* + 154.0) hartree, of the lowest singlet [panels (a) and (b)] and triplet [panels (c) and (d)] states of cyclobutadiene, as described by the cc-pVDZ basis set, and the Δ*E*_S–T_ gaps between them [panels (e) and (f)] toward their CCSDT counterparts with the CIPSI wave function termination parameter *N*_det(in)_ at selected values of the dimensionless variable λ defining the automerization coordinate via the interpolation formula given by eq 2. The CC­(*P*) results are reported in panels (a), (c), and (e). Panels (b), (d), and (f) show the corresponding CC­(*P*;*Q*) data.

Let us first comment on the calculations using *N*_det(in)_ = 1, which help us appreciate the need for including the leading triply excited determinants in the *P* spaces employed in the CC­(*P*) and CC­(*P*;*Q*) computations, especially when *T*_3_ effects and the coupling between *T*_1_ and *T*_2_ clusters and their higher-rank *T*_3_ counterpart become significant. As explained in Section 2, when *N*_det(in)_ = 1, the CIPSI-driven CC­(*P*) and CC­(*P*;*Q*) approaches become equivalent to CCSD and CR-CC­(2,3), respectively, i.e., one solves the CCSD equations for *T*_1_ and *T*_2_ clusters, as if they were decoupled from their higher-rank *T*_3_ counterpart, and corrects the resulting CCSD energies for the effects of connected triples using the CR-CC­(2,3) method. Upon examining the *N*_det(in)_ = 1 CC­(*P*) values in Table 1 (cf., also, Figure 2), we observe that the *T*_3_ correlation effects characterizing the lowest-energy ^1^ *A*_*g*_(*D*_2h_) potential, estimated by forming differences between the CCSDT and CCSD energies, are not only large but also dramatically changing along the automerization coordinate, from −26.827 millihartree for the λ = 0 R species to −47.979 millihartree for the λ = 1 TS structure when the cc-pVDZ basis set is employed. As indicated by the *N*_det(in)_ = 1 CC­(*P*;*Q*) results shown in Table 1 and Figure 2, the incorporation of *T*_3_ correlations using the noniterative CR-CC­(2,3) corrections to the CCSD energies helps, reducing the 26.827, 27.964, 29.667, 32.473, 37.662, and 47.979 millihartree errors relative to CCSDT obtained at λ = 0, 0.2, 0.4, 0.6, 0.8, and 1 with CCSD to 0.848, 1.253, 2.021, 3.582, 7.008, and 14.636 millihartree, respectively, but substantial discrepancies between the CR-CC­(2,3) and CCSDT data, especially in the neighborhood of the barrier region, where they are as large as 7–15 millihartree when λ ∈ [0.8,1], remain. As a result, the quality of the ^1^ *A*_*g*_(*D*_2h_) potential obtained in the CR-CC­(2,3) calculations, which is characterized by a large, 13.788 millihartree, nonparallelity error (NPE) relative to its CCSDT parent and which is shown in Figure 5b, is poor. Failure of CR-CC­(2,3) and, as demonstrated, for example, in refs 109 and 164 , of other noniterative triples corrections to CCSD, including CCSD(T)^109,164^ and CCSD(2)_T_,^109^ to accurately describe the lowest ^1^ *A*_*g*_(*D*_2h_) state of cyclobutadiene in the automerization barrier region is, in significant part, a consequence of the inability of all such methods to capture the coupling of the lower-rank *T*_1_ and *T*_2_ clusters with *T*_3_, which in the vicinity of the TS geometry, where *T*_3_ effects are large and nonperturbative, is big enough to substantially alter *T*_1_ and *T*_2_ amplitudes compared to their CCSD values. This means that to improve the quality of the ^1^ *A*_*g*_(*D*_2h_) potential obtained with CR-CC­(2,3), one should relax *T*_1_ and *T*_2_ clusters, adjusting them to the presence of *T*_3_ correlations, prior to determining noniterative triples corrections. The CIPSI-driven CC­(*P*;*Q*) methodology allows us to do it in a computationally efficient manner, avoiding expensive CCSDT iterations, by incorporating the leading triply excited determinants identified with the help of the CIPSI runs using sufficiently large *N*_det(in)_ > 1 values into the underlying *P* spaces and correcting the resulting CC­(*P*) energies for the remaining *T*_3_ effects using the δ­(*P*;*Q*) corrections.

The situation with the lowest-energy ^3^ *B*_1*g*_(*D*_2h_) potential is different. In this case, as shown in Table 2 and Figure 3, the *T*_3_ correlation effects, estimated, after subtracting the CCSD energies from their CCSDT counterparts obtained with cc-pVDZ, at about (−25) to (−24) millihartree, barely vary with the automerization coordinate λ and are accurately described by the CR-CC­(2,3) approach, which reproduces the parent CCSDT energetics to within 60 microhartree across the entire ^3^ *B*_1*g*_(*D*_2h_) PEC. Because of this very different behavior of CR-CC­(2,3) compared to the lowest-energy singlet potential, which can be seen by comparing the *N*_det(in)_ = 1 CC­(*P*;*Q*) PECs in the (b) and (d) panels of Figure 5, one ends up with a highly unbalanced description of the lowest singlet and triplet states of cyclobutadiene by the CR-CC­(2,3) method, especially in the vicinity of the barrier on the ^1^ *A*_*g*_(*D*_2h_) potential. This is reflected in the errors characterizing the singlet–triplet gap values obtained with CR-CC­(2,3), relative to CCSDT, which in the calculations using the cc-pVDZ basis set grow from 0.553 kcal/mol at λ = 0 to 9.222 kcal/mol when the λ = 1 square TS structure is considered [see the *N*_det(in)_ = 1 CC­(*P*;*Q*) values of Δ*E*_S–T_ in Table 3; cf., also, Figures 4 and 5f]. Once again, the main problem resides in the neglect of the coupling between *T*_1_ and *T*_2_ clusters and their higher-rank *T*_3_ counterpart in the CR-CC­(2,3) approach, which results in a poor description of the ^1^ *A*_*g*_(*D*_2h_) potential in the vicinity of the TS geometry that propagates into the similarly poor Δ*E*_S–T_ gap values. To bring the results closer to those obtained with CCSDT, the input variable *N*_det(in)_ that controls the CIPSI runs preceding the CC­(*P*) and CC­(*P*;*Q*) steps must be increased. The results of the CIPSI-driven CC­(*P*)/cc-pVDZ and CC­(*P*;*Q*)/cc-pVDZ computations for the lowest ^1^ *A*_*g*_(*D*_2h_) and ^3^ *B*_1*g*_(*D*_2h_) potentials of cyclobutadiene and the gap between them using representative *N*_det(in)_ > 1 values are discussed next.

As shown in Table 1 and Figures 2 and 5b, the CC­(*P*;*Q*) computations for the lowest ^1^ *A*_*g*_(*D*_2h_) state, using CIPSI Hamiltonian diagonalizations to identify the leading triply excited determinants for inclusion in the underlying *P* spaces, display fast convergence toward CCSDT with *N*_det(in)_, independent of the value of λ. In the case of the calculations performed using the cc-pVDZ basis set discussed here, with as little as 101,361–196,965 *S*_*z*_ = 0 determinants of the *A*_*g*_(*D*_2h_) symmetry in the terminal |Ψ^(CIPSI)^⟩ wave functions generated by the inexpensive CIPSI runs using *N*_det(in)_ = 100,000, which capture tiny fractions, on the order of 0.1–0.2%, of the 14,483,876 *A*_*g*_(*D*_2h_)-symmetric *S*_*z*_ = 0 triples, the CC­(*P*;*Q*) method reduces the 0.848, 1.253, 2.021, 3.582, 7.008, and 14.636 millihartree errors relative to CCSDT obtained at λ = 0, 0.2, 0.4, 0.6, 0.8, and 1 with CR-CC­(2,3) to 0.431, 0.552, 0.776, 1.235, 0.628, and 3.539 millihartree, respectively. With the relatively small additional effort corresponding to *N*_det(in)_ = 250,000, which results in 394,080–449,753 *S*_*z*_ = 0 determinants of the *A*_*g*_(*D*_2h_) symmetry in the final CIPSI diagonalization spaces and only 0.5–0.6% of all triples in the underlying *P* spaces, the differences between the CC­(*P*;*Q*) and CCSDT energies of the lowest singlet state of cyclobutadiene at λ = 0, 0.2, 0.4, 0.6, 0.8, and 1 decrease to 0.278, 0.272, 0.271, 0.300, 0.336, and 0.458 millihartree, respectively. Clearly, these are massive error reductions compared to the CR-CC­(2,3) computations, especially in the barrier region, which highlight the effectiveness of our CIPSI-driven CC­(*P*;*Q*) strategy and the importance of relaxing *T*_1_ and *T*_2_ amplitudes in the presence of the leading *T*_3_ contributions compared to their CCSD values prior to determining the noniterative corrections for the remaining *T*_3_ effects. Similar comments apply to the improvements in the troublesome 13.788 millihartree NPE relative to CCSDT characterizing the ^1^ *A*_*g*_(*D*_2h_) potential obtained in the CR-CC­(2,3)/cc-pVDZ calculations offered by the CIPSI-driven CC­(*P*;*Q*) runs. When the CC­(*P*;*Q*)/cc-pVDZ approach using *N*_det(in)_ = 100,000 is employed, the NPE relative to CCSDT characterizing the resulting ^1^ *A*_*g*_(*D*_2h_) potential becomes 3.108 millihartree, which is a reduction of the corresponding CR-CC­(2,3) NPE value by a factor of 4.4. The *N*_det(in)_ = 250,000 CC­(*P*;*Q*) computations, which extract the lists of triples from the relatively inexpensive Hamiltonian diagonalizations in spaces that in the case of the cc-pVDZ basis set are 32–37 times smaller than the number of *T*_3_ amplitudes used by CCSDT, reduce the 13.788 millihartree NPE characterizing the lowest-energy ^1^ *A*_*g*_(*D*_2h_) potential obtained with CR-CC­(2,3)/cc-pVDZ, relative to its CCSDT counterpart, to an impressively small value of 0.187 millihartree. This is a 74-fold reduction in NPE compared to CR-CC­(2,3).

It is clear from Table 1 and Figures 2 and 5b that the convergence of the lowest-energy ^1^ *A*_*g*_(*D*_2h_) potentials resulting from the CIPSI-driven CC­(*P*;*Q*) calculations toward their CCSDT parent, including the challenging barrier region, with the CIPSI wave function termination parameter *N*_det(in)_, with the number of determinants in the final Hamiltonian diagonalization space used to determine |Ψ^(CIPSI)^⟩ [*N*_det(out)_], and with the fraction of triply excited determinants in the *P* space captured by CIPSI is very fast, but one cannot say the same about the uncorrected CC­(*P*) energies. As shown in Table 1 and Figures 2 and 5a, and in line with the formal analysis in Section 2, the CC­(*P*) energies improve the CCSD results and converge toward CCSDT, but they do it at a much slower rate than their δ­(*P*;*Q*)-corrected CC­(*P*;*Q*) counterparts. For instance, the CIPSI-driven CC­(*P*)/cc-pVDZ computations for the lowest singlet state of cyclobutadiene using *N*_det(in)_ = 100,000 reduce the 26.827, 27.964, 29.667, 32.473, 37.662, and 47.979 millihartree errors relative to CCSDT obtained at λ = 0, 0.2, 0.4, 0.6, 0.8, and 1 with CCSD/cc-pVDZ and the associated NPE value of 21.152 millihartree to 22.183, 23.029, 23.947, 25.279, 21.842, 32.125, and 10.283 millihartree, respectively. This should be compared to the much smaller error and NPE values characterizing the corresponding CC­(*P*;*Q*)/cc-pVDZ calculations, which are 0.431–3.539 and 3.108 millihartree, respectively. The analogous CC­(*P*) calculations using *N*_det(in)_ = 250,000, where the errors characterizing the δ­(*P*;*Q*)-corrected CC­(*P*;*Q*) energies in the entire λ = 0–1 region and the overall NPE relative to CCSDT are already at the level of 0.2–0.5 millihartree, produce the 17.137–18.165 millihartree errors and the NPE of 1.028 millihartree. Even with the largest *N*_det(in)_ value considered in this study, of 10,000,000, the 7.252–10.238 millihartree differences between the CC­(*P*)/cc-pVDZ and CCSDT/cc-pVDZ energies of the lowest ^1^ *A*_*g*_(*D*_2h_) state of cyclobutadiene in the λ = 0–1 region and the NPE of 2.986 millihartree that characterizes the resulting CC­(*P*) potential relative to its CCSDT parent remain. All of this implies that while relaxing *T*_1_ and *T*_2_ amplitudes in the presence of the leading triples is important, correcting the CC­(*P*) energies for the remaining *T*_3_ effects, which the CC­(*P*) computations using the *P* spaces generated with the help of CIPSI do not describe, is critical to reach submillihartree accuracy levels relative to CCSDT with small fractions of triples in these spaces. We observed a similar behavior in the semistochastic, CIQMC- and CCMC-driven,^32,122–124^ and adaptive^126^ CC­(*P*) and CC­(*P*;*Q*) calculations, although based on the numerical evidence that we have generated to date, the CIPSI-driven CC­(*P*;*Q*) methodology investigated in this study and its recently formulated adaptive analog seem to be more effective in converging the target CC (in most of our work to date, CCSDT) energetics than their semistochastic counterparts. This suggests that the sequences of Hamiltonian diagonalizations utilized in the CIPSI-driven CC­(*P*) and CC­(*P*;*Q*) computations and the moment expansions defining the δ­(*P*;*Q*) corrections that are used to construct excitation spaces in the adaptive CC­(*P*) and CC­(*P*;*Q*) runs are more efficient in identifying the leading higher-than-doubly excited determinants for inclusion of the underlying *P* spaces than the CIQMC/CCMC wave function propagations, although this topic needs to be explored further and we will return to it in the future.

As shown in Table 2 and Figures 3 and 5c,d, many of the above observations apply to the CIPSI-driven CC­(*P*) and CC­(*P*;*Q*) calculations for the lowest-energy ^3^ *B*_1*g*_(*D*_2h_) potential, but, given the fact that the CR-CC­(2,3) approach is already very accurate in this case, producing errors relative to CCSDT that in absolute value do not exceed 60 microhartree when the cc-pVDZ basis set is employed, the CC­(*P*;*Q*) computations using *N*_det(in)_ > 1 offer no obvious advantages over CR-CC­(2,3). Nonetheless, it is reassuring that, in analogy to the *A*_*g*_(*D*_2h_)-symmetric singlet ground state, the differences between the energies of the ^3^ *B*_1*g*_(*D*_2h_) state obtained in the CC­(*P*) computations using CIPSI Hamiltonian diagonalizations to create lists of the leading triply excited determinants for inclusion in the underlying *P* spaces and their CCSDT counterparts decrease as *N*_det(in)_ increases, independent of λ. It is also encouraging that the δ­(*P*;*Q*) corrections are as effective in bringing the CC­(*P*) energies to a virtually perfect agreement with the parent CCSDT data as in the case of the CIPSI-driven CC­(*P*;*Q*)/cc-pVDZ calculations for the lowest ^1^ *A*_*g*_(*D*_2h_) potential using *N*_det(in)_ ≥ 250,000. By inspecting the CC­(*P*;*Q*) column in Table 2, one may get the impression that the incorporation of the triply excited determinants identified by the CIPSI runs using increasingly large *N*_det(in)_ values in the preceding CC­(*P*) steps worsens the CR-CC­(2,3) results for the lowest ^3^ *B*_1*g*_(*D*_2h_) state, which correspond to *N*_det(in)_ = 1, but reading Table 2 in this way would be misleading. Indeed, in single-reference situations, such as that created by the lowest ^3^ *B*_1*g*_(*D*_2h_) state of cyclobutadiene, where many-electron correlation effects are essentially only dynamical, the triples correction of CR-CC­(2,3) [similarly to CCSD­(T)] often overshoots the parent CCSDT energies (slightly). Once one starts adding triply excited determinants to the *P* space, the CC­(*P*;*Q*) energies initially go up, becoming upper bounds to their CCSDT counterparts, but when the fraction of triples in the *P* space is large enough, the differences between the CC­(*P*;*Q*) and CCSDT energies decrease, steadily approaching 0. We see some of this behavior in Table 2, but we have to keep in mind that the *P* spaces used in our CIPSI-driven CC­(*P*;*Q*) calculations use small fractions of triples, so that the residual, ∼0.1 millihartree, errors relative to CCSDT remain. What is most important here is that the CC­(*P*;*Q*) computations for the lowest triplet potential of cyclobutadiene using *N*_det(in)_ > 1 reported in Table 2 and Figures 3 and 5d do not substantially alter the already excellent CR-CC­(2,3) energetics. This allows us to conclude that the coupling of the lower-rank *T*_1_ and *T*_2_ clusters with their higher-rank *T*_3_ counterpart is negligible in this case and the relaxation of the CCSD values of *T*_1_ and *T*_2_ amplitudes by including some triples in the iterative CC­(*P*) steps is not necessary for obtaining high-accuracy CC­(*P*;*Q*) results.

Having demonstrated the excellent performance of the CIPSI-driven CC­(*P*;*Q*) approach in accurately approximating the lowest-energy ^1^ *A*_*g*_(*D*_2h_) and ^3^ *B*_1*g*_(*D*_2h_) potentials of cyclobutadiene along its automerization coordinate obtained with CCSDT, we now turn to the CC­(*P*) and CC­(*P*;*Q*) singlet–triplet gaps and their dependence on λ and *N*_det(in)_ examined, using the cc-pVDZ basis, in Table 3 and Figures 4, 5e [the uncorrected CC­(*P*) energetics], and 5f [the CC­(*P*;*Q*) results]. As pointed out above, to obtain accurate Δ*E*_S–T_ values for cyclobutadiene in the vicinity of the barrier region, one has to balance significant nondynamical correlations associated with the multiconfigurational singlet state, which manifest themselves in massive *T*_3_ clusters that are strongly coupled to the one- and two-body components of *T*, with predominantly dynamical correlations characterizing the lowest triplet state that result in the generally smaller *T*_3_ contributions having minimal effect on *T*_1_ and *T*_2_ amplitudes. The singlet–triplet gap values reported in Table 3 and Figures 4 and 5e,f clearly show that neither the CCSD approach nor the CR-CC­(2,3) triples correction to CCSD, which are equivalent to the CIPSI-driven CC­(*P*) and CC­(*P*;*Q*) calculations using *N*_det(in)_ = 1, can do this. Both of these methods struggle with achieving a balanced description of the ^1^ *A*_*g*_(*D*_2h_) and ^3^ *B*_1*g*_(*D*_2h_) states of cyclobutadiene as λ approaches 1, producing errors relative to CCSDT that in the calculations using the cc-pVDZ basis set are as large as 5.261 and 2.282 kcal/mol, respectively, at λ = 0.6, where the CCSDT value of Δ*E*_S–T_ is −10.295 kcal/mol, 8.589 and 4.434 kcal/mol, respectively, at λ = 0.8, where Δ*E*_S–T_ obtained with CCSDT is −5.804 kcal/mol, and 15.120 and 9.222 kcal/mol at λ = 1, where the CCSDT result for Δ*E*_S–T_ is −4.783 kcal/mol. These large error values in the singlet–triplet gaps resulting from the CCSD and CR-CC­(2,3) computations in the barrier region are a consequence of the dramatic increase in the magnitude of *T*_3_ effects characterizing the ^1^ *A*_*g*_(*D*_2h_) state and a rapidly deteriorating description of this state by both CCSD and CR-CC­(2,3) as λ → 1, seen in Table 1 and Figures 2 and 5a,b, as opposed to the nearly constant *T*_3_ contributions and a virtually perfect agreement between the CR-CC­(2,3) and CCSDT ^3^ *B*_1*g*_(*D*_2h_) potentials in the entire λ = 0–1 region shown in Table 2 and Figures 3 and 5d [while quantitatively inaccurate, the shape of the ^3^ *B*_1*g*_(*D*_2h_) potential obtained with CCSD, shown in Figure 5c, is qualitatively correct too]. In analogy to the previously discussed calculations for the lowest-energy ^1^ *A*_*g*_(*D*_2h_) potential, in order to bring the above errors down, the input parameter *N*_det(in)_, which controls the CIPSI diagonalization sequences preceding the CC­(*P*) and CC­(*P*;*Q*) computations, must be increased, so that the *P* spaces used in these computations are augmented with the leading triply excited determinants, the CCSD values of *T*_1_ and *T*_2_ amplitudes, used in CR-CC­(2,3), are properly relaxed, and the quality of the δ­(*P*;*Q*) corrections that capture the remaining *T*_3_ effects improves.

This is precisely what we observe in the CIPSI-driven CC­(*P*) and CC­(*P*;*Q*) calculations of the singlet–triplet gaps, especially the latter ones, reported in Table 3 and Figures 4 and 5e,f. Indeed, the CC­(*P*;*Q*) approach using the cc-pVDZ basis and *N*_det(in)_ = 100,000, which relies on small CIPSI diagonalization spaces [small *N*_det(out)_ values], whose dimensionalities are about 1% of the 14,483,876 *A*_*g*_(*D*_2h_)-symmetric *S*_*z*_ = 0 and 14,339,992 *B*_1*g*_(*D*_2h_)-symmetric *S*_*z*_ = 1 triply excited amplitudes involved in the parent full CCSDT computations, and which employs even smaller *P* spaces having only 0.1–0.6% of all triples, reduces the 0.553, 0.813, 1.300, 2.282, 4.434, and 9.222 kcal/mol differences between the CR-CC­(2,3)/cc-pVDZ and CCSDT/cc-pVDZ Δ*E*_S–T_ values at λ = 0, 0.2, 0.4, 0.6, 0.8, and 1 to 0.253, 0.301, 0.485, 0.784, 0.398, and 2.227 kcal/mol, respectively. When *N*_det(in)_ is increased to 250,000, where the numbers of determinants included in the final CIPSI diagonalizations preceding the CC­(*P*) and CC­(*P*;*Q*) steps are still only ∼2–3% of all *T*_3_ amplitudes used in the target CCSDT/cc-pVDZ calculations for the ^1^ *A*_*g*_(*D*_2h_) and ^3^ *B*_1*g*_(*D*_2h_) states, and where the resulting *P* spaces contain only 0.5–0.9% of all triples, the errors in the CC­(*P*;*Q*) Δ*E*_S–T_ gaps relative to their CCSDT counterparts obtained at the above values of λ with the cc-pVDZ basis set decrease even more, to 0.112, 0.108, 0.128, 0.128, 0.152, and 0.263 kcal/mol, respectively, bringing the CC­(*P*;*Q*) and CCSDT results to a virtually perfect agreement, while reducing a computational effort compared to the CCSDT runs by orders of magnitude. As in the previously discussed results for the lowest singlet and triplet states of cyclobutadiene, especially for the challenging, *A*_*g*_(*D*_2h_)-symmetric, singlet ground state, the δ­(*P*;*Q*) corrections play a major role in the observed error reductions, substantially improving the CC­(*P*) Δ*E*_S–T_ values, but, unlike in the CC­(*P*) calculations of the ^1^ *A*_*g*_(*D*_2h_) and ^3^ *B*_1*g*_(*D*_2h_) potentials, the singlet–triplet gaps obtained with the uncorrected CC­(*P*) approach using relatively small CIPSI diagonalization spaces and tiny fractions of all triples in the associated *P* spaces can be quite accurate in their own right, reproducing the CCSDT values of Δ*E*_S–T_ across the entire λ = 0–1 region to within ∼1–2 kcal/mol when *N*_det(in)_ ≳ 250,000 and the cc-pVDZ basis set is employed. Clearly, this is a lot better than the 5.261, 8.589, and 15.120 kcal/mol errors relative to CCSDT obtained at λ = 0.6, 0.8, and 1, respectively, with CCSD using the same basis, demonstrating that the relaxation of *T*_1_ and *T*_2_ amplitudes in the presence of the leading *T*_3_ contributions in the CIPSI-driven CC­(*P*) computations using sufficiently large *N*_det(in)_ values results in a more balanced description of the many-electron correlation effects in the ^1^ *A*_*g*_(*D*_2h_) and ^3^ *B*_1*g*_(*D*_2h_) states, especially when λ approaches 1, although, by inspecting Tables 1–3 and Figures 2–4, we can also see that much of the improvement in the CCSD Δ*E*_S–T_ data offered by the CC­(*P*) approach originates from error cancellations between the ^1^ *A*_*g*_(*D*_2h_) and ^3^ *B*_1*g*_(*D*_2h_) CC­(*P*) energies. In the case of the CIPSI-driven CC­(*P*;*Q*) computations, we do not have to count on error cancellations, since both the total electronic energies of the lowest singlet and triplet states of cyclobutadiene at various values of λ and the gaps between them rapidly converge toward their CCSDT parents with *N*_det(in)_. The δ­(*P*;*Q*) corrections, in addition to being highly effective in improving the CC­(*P*) energies of the ^1^ *A*_*g*_(*D*_2h_) and ^3^ *B*_1*g*_(*D*_2h_) states and the associated Δ*E*_S–T_ values, are also very helpful in curing the nonsystematic error patterns in the singlet–triplet gaps observed in the CC­(*P*) calculations in Table 3 and Figure 4 as *N*_det(in)_ increases, where the differences between the CC­(*P*) and CCSDT values of Δ*E*_S–T_ go up and down or oscillate. This does not happen in the CC­(*P*;*Q*) calculations, where the resulting singlet–triplet gaps approach their CCSDT parents systematically and very fast as *N*_det(in)_ is made larger, independent of λ. This is yet another demonstration of the ability of the CIPSI-driven CC­(*P*;*Q*) approach to provide a highly accurate and well-balanced description of the many-electron correlation effects characterizing the lowest-energy singlet and triplet states of cyclobutadiene along its automerization coordinate, which the conventional CC methods, such as CCSD, CR-CC­(2,3), and other noniterative triple corrections to CCSD, cannot provide as one approaches the barrier region.

Given our generally positive experiences with the active-orbital-based variant of the CC­(*P*;*Q*) methodology abbreviated as CC­(t;3),^22,105,109,115,116,121,127^ which corrects the CCSDt energetics for those *T*_3_ correlations that are not captured by the active-space CCSDt approach, it is interesting to compare the lowest ^1^ *A*_*g*_(*D*_2h_) and ^3^ *B*_1*g*_(*D*_2h_) potentials of cyclobutadiene and the gap between them obtained in the CIPSI-driven CC­(*P*) and CC­(*P*;*Q*) calculations with their CCSDt and CC(t;3) counterparts. This is done in Figure 6, which compares the PECs corresponding to the lowest singlet and triplet states of cyclobutadiene, as described by the cc-pVDZ basis set, along the *D*_2h_-symmetric automerization pathway defined by eq 2 resulting from the CIPSI-based CC­(*P*) and CC­(*P*;*Q*) calculations employing *N*_det(in)_ = 250,000 with the analogous potentials generated with the active-orbital-based CCSDt and CC­(t;3) methods and full CCSDT. The numerical data used to construct the CCSDt and CC­(t;3) PECs shown in Figure 6 and to determine the gap between them can be found in Tables S1–S3 of the Supporting Information. We recall that in the language of the CC­(*P*) and CC­(*P*;*Q*) formalisms, the *P* space adopted in the CCSDt iterations consists of all singly and doubly excited determinants and the subset of triply excited determinants that fit the formula |Φ_*ij* **K**_^**A** *bc*^⟩, where *i*, *j* (*b*, *c*) designate the spin-orbitals occupied (unoccupied) in the reference determinant |Φ⟩ and **K** (**A**) are the occupied (unoccupied) spin-orbitals around the Fermi level belonging to the user-specified active set,^73,128–136^ and the complementary *Q* space needed to determine the CC­(t;3) correction to CCSDt using eq 7 is spanned by the remaining triply excited determinants |Φ_*ijk*_^*abc*^⟩ outside the |Φ_*ij* **K**_^**A** *bc*^⟩ set. In the specific case of the CCSDt and CC­(t;3) computations reported in Figure 6 and Tables S1–S3 of the Supporting Information, the active space defining the subsets of triply excited determinants included in the CCSDt calculations preceding the determination of the noniterative CC­(t;3) corrections consisted of two orbitals of cyclobutadiene that correlate with the valence *e*_*g*_ shell of the *D*_4h_-symmetric TS (λ = 1) structure, meaning the highest occupied and lowest unoccupied RHF orbitals for the lowest ^1^ *A*_*g*_(*D*_2h_) state and the two singly occupied ROHF orbitals in the case of the lowest ^3^ *B*_1*g*_(*D*_2h_) state. As explained in the Supporting Information (see footnotes “b” in Tables S1 and S2), with these choices of active orbitals, the *P* space used in the CCSDt computations for the lowest singlet state contained 1.5% of the *S*_*z*_ = 0 triples of the *A*_*g*_(*D*_2h_) symmetry involved in the parent full CCSDT work, whereas that for the lowest triplet state contained 1.1% of all *S*_*z*_ = 1 *B*_1*g*_(*D*_2h_)-symmetric triples. These percentages should be compared to 0.5–0.6% and 0.6–0.9% of triples of the *S*_*z*_ = 0 *A*_*g*_(*D*_2h_) and *S*_*z*_ = 1 *B*_1*g*_(*D*_2h_) symmetries, respectively, captured by the CIPSI runs preceding the CC­(*P*) and CC­(*P*;*Q*) calculations with *N*_det(in)_ = 250,000, which, as shown in Figure 6 (cf., also, Tables S1–S3 of the Supporting Information and Tables 1–3), produce the results that are in generally very good agreement with their CCSDt and CC­(t;3) counterparts. One might argue that in spite of having a somewhat larger fraction of triply excited determinants in the *P* spaces employed in the CCSDt calculations compared to the numbers of triples identified by the *N*_det(in)_ = 250,000 CIPSI runs, the CC­(t;3) results for the lowest-energy ^3^ *B*_1*g*_(*D*_2h_) state reported in Table S2 of the Supporting Information are less accurate than those obtained with the CIPSI-based CC­(*P*;*Q*) approach using *N*_det(in)_ = 250,000 (or even smaller *N*_det(in)_ values) shown in Table 2. One might also argue that with the exception of the TS region, the usage of a larger fraction of triples in the *P* spaces employed in the CCSDt computations for the lowest ^1^ *A*_*g*_(*D*_2h_) state compared to the numbers of the triples identified by CIPSI using *N*_det(in)_ = 250,000 does not translate into substantial improvements in the CIPSI-driven CC­(*P*;*Q*) results based on this *N*_det(in)_ value by CC­(t;3) (cf. Table S1 of the Supporting Information and Table 1). None of this, however, alters our conclusion regarding the generally good agreement between the CIPSI-driven CC­(*P*) and CC­(*P*;*Q*) calculations for the lowest singlet and triplet potentials of cyclobutadiene, as described by the cc-pVDZ basis, and the gap between them obtained with *N*_det(in)_ = 250,000 and their CCSDt and CC­(t;3) counterparts, and none of this is surprising. Indeed, our choice of active orbitals in the CCSDt and CC­(t;3) calculations discussed here reflects on the multiconfigurational character of the singlet ground state in the vicinity of the λ = 1 TS region, but is not necessarily best for the lowest triplet state, which is dominated by dynamical correlations at all values of λ, or the singlet ground state as λ → 0, where dynamical correlations dominate as well. Furthermore, unlike in the CCSDt case, the lists of triply excited determinants included in the *P* spaces adopted in the CIPSI-driven CC­(*P*) calculations preceding the determination of the CC­(*P*;*Q*) corrections may vary with the nuclear geometry (adjusting to the wave function content as the nuclear geometry changes), so they tend to be more compact than those employed by CCSDt if the active-orbital-based CC­(t;3) and CIPSI-based CC­(*P*;*Q*) computations become similarly accurate [cf. ref 127 for the analogous comments regarding the adaptive CC­(*P*;*Q*) framework vs CC­(t;3)]. Having said all this, the similarity between the PECs characterizing the lowest singlet and triplet states of cyclobutadiene, as described by the cc-pVDZ basis, along its automerization pathway resulting from the CIPSI-driven CC­(*P*;*Q*) calculations using *N*_det(in)_ = 250,000 and their counterparts obtained with the CC­(t;3) approach using a chemically motivated active space consisting of two valence orbitals, combined with the observation that these two independent computations accurately approximate the parent CCSDT data, is reassuring. It demonstrates that while there may be some differences between the subsets of triply excited determinants identified with the help of active orbitals following the CCSDt recipe and those extracted from the CIPSI runs, these differences are relatively small if the terminal diagonalization space used by the CIPSI approach that drives the CC­(*P*) and CC­(*P*;*Q*) computations is sufficiently large and the active space used to set up the CCSDt calculations preceding the determination of the CC­(t;3) corrections is reasonable. In other words, both CCSDt and CIPSI are capable of capturing the leading triples for inclusion in the *P* spaces used by the CC­(*P*) and CC­(*P*;*Q*) calculations, but CIPSI allows us to do it in a more black-box fashion without having to resort to user-defined active orbitals, which is certainly appealing. This remark is in line with one of our earlier studies, reported in ref 123, where we compared the manifolds of triply excited determinants captured in the context of the semistochastic CC­(*P*;*Q*) considerations by CIQMC with those defined by the |Φ_*ij* **K**_^**A** *bc*^⟩ formula of CCSDt.

**Figure 6:**
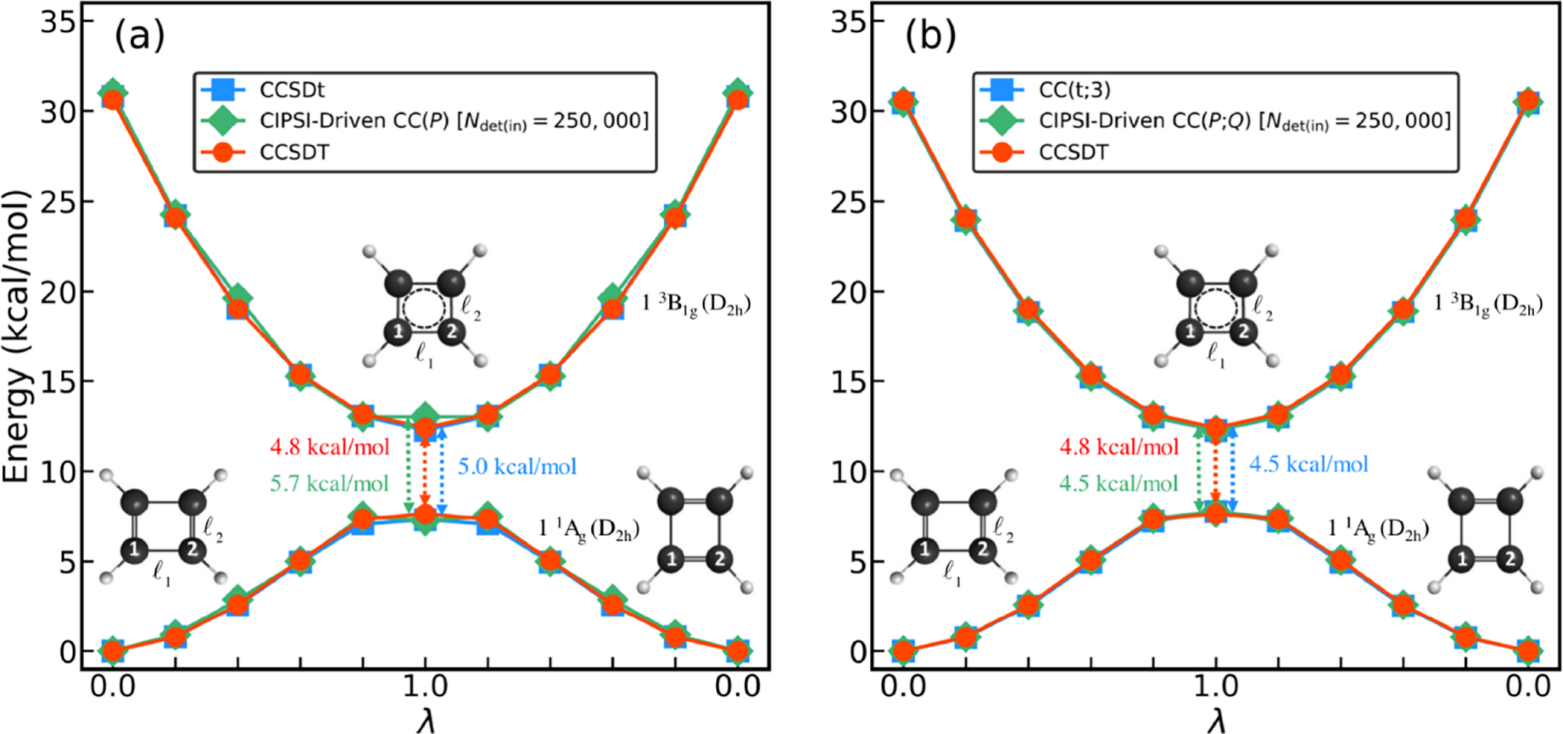
The PECs (in kcal/mol) characterizing the lowest-energy singlet and triplet states of cyclobutadiene, as described by the cc-pVDZ basis set, along the *D*_2h_-symmetric automerization pathway, defined using the interpolation formula given by eq 2 and parametrized by the dimensionless variable λ. Panel (a) presents the active-orbital-based CCSDt (blue solid squares and lines), CIPSI-driven CC­(*P*) (green solid diamonds and lines), and parent CCSDT data (red solid circles and lines), and panel (b) shows the corresponding CC­(t;3) (blue solid squares and lines) and CIPSI-driven CC­(*P*;*Q*) (green solid diamonds and lines) results, in addition to the full CCSDT energetics (red solid circles and lines). The active space defining the subsets of triply excited determinants included in the CCSDt and CC­(t;3) calculations consisted of two orbitals of cyclobutadiene that correlate with the valence *e*_*g*_ shell of the *D*_4h_-symmetric TS (λ = 1) structure, whereas the lists of triples entering the *P* spaces employed in the CIPSI-based CC­(*P*) and CC­(*P*;*Q*) computations were extracted from the terminal wave functions |Ψ^(CIPSI)^⟩ obtained with *N*_det(in)_ = 250,000. For each of the methods in panels (a) and (b), the energy of the singlet ground state at the reactant (R, λ = 0) geometry is set to 0. The numbers in the middle of each panel, colored in the same way as the corresponding PECs, are the unsigned values of the singlet–triplet gaps determined at the λ = 1 TS structure.

With the exception of specific errors relative to CCSDT at various values of *N*_det(in)_, much of the above discussion applies to basis sets larger than cc-pVDZ. In fact, for larger basis sets, the benefits of using the CIPSI-driven CC­(*P*;*Q*) approach to obtain the near-CCSDT energetics at small fractions of the computational costs are expected to be even greater than those observed for cc-pVDZ since one can continue using relatively small CIPSI diagonalization spaces to determine the subsets of triples entering the CC­(*P*) computations, whereas the manifolds of all triply excited determinants and amplitudes used by full CCSDT, which for a given number of electrons scale as cube of the number of unoccupied orbitals, grow with the size of the one-electron basis very fast. This is illustrated in Tables 4–6, where we report the CIPSI-driven CC­(*P*) and CC­(*P*;*Q*) calculations using the cc-pVTZ basis set, along with the associated variational (*E*_var_) and perturbatively corrected (*E*_var_ + Δ*E* ^(2)^ and *E*_var_ + Δ*E*_r_^(2)^) CIPSI energies, for the lowest ^1^ *A*_*g*_(*D*_2h_) and ^3^ *B*_1*g*_(*D*_2h_) states of cyclobutadiene and the gaps between them at the key R (λ = 0) and TS (λ = 1) geometries. In this case, the CC­(*P*;*Q*) computations result in small ∼0.4–0.6 millihartree, ∼0.1 millihartree, and ∼0.1–0.3 kcal/mol errors relative to CCSDT for the lowest singlet state, lowest triplet state, and Δ*E*_S–T_, respectively, when *N*_det(in)_ = 1,000,000, i.e., when the *N*_det(in)_ value is only 4 times larger than that leading to similar accuracies in the CC­(*P*;*Q*)/cc-pVDZ calculations, but the total numbers of the *A*_*g*_(*D*_2h_)-symmetric *S*_*z*_ = 0 and *B*_1*g*_(*D*_2h_)-symmetric *S*_*z*_ = 1 triply excited amplitudes used by the parent CCSDT/cc-pVTZ approach, which are 260,030,720 and 258,073,116, respectively, exceed those employed by its CCSDT/cc-pVDZ counterpart by a factor of 18. As a result, the convergence of the CIPSI-driven CC­(*P*;*Q*) energetics toward CCSDT resulting from the calculations employing the cc-pVTZ basis set reported in Tables 4–6 is impressive. For example, as shown in Table 4, the CC­(*P*;*Q*)/cc-pVTZ calculation for the lowest-energy singlet state at the challenging TS structure using the relatively small *N*_det(in)_ value of 1,000,000, which relies on the CIPSI diagonalization space whose dimensionality [*N*_det(out)_] is a tiny 0.5% of the *S*_*z*_ = 0 triples of the *A*_*g*_(*D*_2h_) symmetry involved in the parent full CCSDT/cc-pVTZ work, and which employs an even tinier *P* space having only 0.1% of all triples in it, reduces the 13.793 millihartree error relative to CCSDT obtained with CR-CC­(2,3) to 0.591 millihartree. Given that, analogous to the cc-pVDZ case, the CC­(*P*;*Q*) approach using the cc-pVTZ basis offers a virtually perfect description of the lowest-energy triplet state, with the error relative to CCSDT obtained with *N*_det(in)_ = 1,000,000 at λ = 1 of only 0.138 millihartree (even though the dimensionality of the associated CIPSI space is a tiny 0.6% of the *B*_1*g*_(*D*_2h_)-symmetric *S*_*z*_ = 1 triply excited amplitudes used by CCSDT and the underlying *P* space contains only 0.2% of all triples), the observed excellent performance of the CIPSI-driven CC­(*P*;*Q*) methodology for the singlet state translates into a highly accurate description of the singlet–triplet gap by CC­(*P*;*Q*)/cc-pVTZ, which reduces the 8.685 kcal/mol error obtained at λ = 1 with CR-CC­(2,3) to 0.285 kcal/mol when *N*_det(in)_ is set to 1,000,000. As one might anticipate in light of the previously discussed CC­(*P*;*Q*) calculations using a smaller cc-pVDZ basis, the errors for the “easier” λ = 0 geometry resulting from the CIPSI-driven CC­(*P*;*Q*)/cc-pVTZ calculations are generally smaller than their λ = 1 counterparts and the results improve with increasing *N*_det(in)_, but it is most encouraging to observe that the CC­(*P*;*Q*)/cc-pVTZ approach is capable of providing an excellent description of the lowest singlet and triplet states of cyclobutadiene and the gap between them at both the R and TS geometries, with errors relative to CCSDT/cc-pVTZ on the order of small fractions of a millihartree or kilocalorie per mole, based on the relatively small CIPSI diagonalization spaces, such as those corresponding to *N*_det(in)_ = 1,000,000, which are only a few times larger than those employed in the similarly well-converged CC­(*P*;*Q*) calculations using cc-pVDZ. In analogy to the cc-pVDZ basis set, it is reassuring to observe the remarkable efficiency of the δ­(*P*;*Q*) correction defined by eq 7 in reducing errors obtained with the uncorrected CC­(*P*) approach at all values of *N*_det(in)_ included in Tables 4–6 when the larger cc-pVTZ basis set is employed. The consistency of our observations regarding the performance of the CIPSI-driven CC­(*P*) and CC­(*P*;*Q*) methods for the lowest singlet and triplet states of cyclobutadiene and the gap between them in calculations using smaller cc-pVDZ and larger cc-pVTZ basis sets is reassuring too.

**Table 4:** Convergence of the CC­(*P*) and CC­(*P*;*Q*) Energies of the Lowest Singlet State of Cyclobutadiene, as Described by the cc-pVTZ Basis Set, Toward CCSDT at the R (λ = 0) and TS (λ = 1) Geometries, Alongside the Associated Variational and Perturbatively Corrected CIPSI Energies

λ	*N*_det(in)_/*N*_det(out)_	% of triples	*E*_var_ ^a^	*E*_var_ + Δ*E* ^(2)^ ^a^	*E*_var_ + Δ*E*_r_^(2)^ ^a^	CC(*P*)^b^	CC(*P*;*Q*)^b^
0	1/1	0.0	702.920^c^	–76.833^d^	151.271	36.016^e^	0.941^f^
	50,000/88,980	0.0	204.608	39.243(330)	44.233(320)	35.977	0.934
	100,000/177,965	0.0	162.217	36.025(252)	38.853(246)	35.450	0.873
	250,000/355,932	0.0	139.878	34.161(203)	36.131(200)	32.851	0.637
	500,000/711,877	0.0	129.692	31.659(193)	33.310(190)	29.254	0.439
	1,000,000/1,423,810	0.1	122.284	28.864(186)	30.323(183)	26.113	0.359
	5,000,000/5,695,067	0.5	108.430	25.507(166)	26.600(163)	20.099	0.291
	10,000,000/11,390,227	0.9	101.366	24.119(154)	25.041(152)	16.896	0.236
1	1/1	0.0	743.761^c^	–84.810^d^	326.149	55.205^e^	13.793^f^
	50,000/83,877	0.0	239.272	68.057(304)	73.536(294)	53.912	12.098
	100,000/169,536	0.0	196.632	64.661(261)	67.837(254)	52.060	10.916
	250,000/339,078	0.0	172.961	60.875(205)	63.128(201)	45.898	7.126
	500,000/678,157	0.0	158.270	53.134(210)	55.059(206)	35.931	2.296
	1,000,000/1,342,908	0.1	139.683	39.101(201)	40.795(197)	26.039	0.591
	5,000,000/5,425,949	0.3	116.366	32.766(167)	33.884(165)	18.615	0.515
	10,000,000/10,744,113	0.6	109.897	31.233(157)	32.198(155)	16.177	0.418

a For each value of λ, the *E*_var_, *E*_var_ + Δ*E* ^(2)^, and *E*_var_ + Δ*E*_r_^(2)^ energies are reported as errors, in millihartree, relative to the extrapolated *E*_var_ + Δ*E*_r_^(2)^ energy found using a linear fit based on the last four *E*_var,*k*_ + Δ*E*_r,*k*_^(2)^ values leading to the largest CIPSI wave function obtained with *N*_det(in)_ = 10,000,000, plotted against the corresponding Δ*E*_r,*k*_^(2)^ corrections, following the procedure described in refs 41, 78, and 163 . The extrapolated *E*_var_ + Δ*E*_r_^(2)^ energies at λ = 0 and 1 are −154.397265(1917) and −154.388862(2773) hartree, respectively, where the error bounds in parentheses correspond to the uncertainty associated with the linear fit. The error bounds for the *E*_var_ + Δ*E* ^(2)^ and *E*_var_ + Δ*E*_r_^(2)^ energies obtained at the various values of *N*_det(in)_ reflect on the semistochastic design of the $V_{\mathrm{ext}}^{\left(k\right)}$ spaces discussed in the main text, but they ignore the uncertainties characterizing the reference *E*_var_ + Δ*E*_r_^(2)^ energies obtained in the above extrapolation procedure.

b The CC­(*P*) and CC­(*P*;*Q*) energies are reported as errors relative to CCSDT, in millihartree. The total CCSDT energies at λ = 0 and 1 are −154.390763 and −154.373902 hartree, respectively.

c Equivalent to RHF.

d Equivalent to the result obtained with the second-order MBPT approach using the Epstein–Nesbet denominator.

e Equivalent to CCSD.

f Equivalent to CR-CC­(2,3).

**Table 5:** Convergence of the CC­(*P*) and CC­(*P*;*Q*) Energies of the Lowest Triplet State of Cyclobutadiene, as Described by the cc-pVTZ Basis Set, Toward CCSDT at the R (λ = 0) and TS (λ = 1) Geometries, Alongside the Associated Variational and Perturbatively Corrected CIPSI Energies

λ	*N*_det(in)_/*N*_det(out)_	% of triples	*E*_var_ ^a^	*E*_var_ + Δ*E* ^(2)^ ^a^	*E*_var_ + Δ*E*_r_^(2)^ ^a^	CC(*P*)^b^	CC(*P*;*Q*)^b^
0	1/1	0	669.778^c^	–108.330^d^	118.961	33.952^e^	–0.023^f^
	50,000/85,613	0.0	248.570	22.169(331)	32.314(316)	33.124	–0.012
	100,000/167,642	0.0	191.558	19.197(329)	24.688(319)	32.708	–0.003
	250,000/341,366	0.1	144.738	16.373(252)	19.315(246)	31.619	–0.006
	500,000/684,969	0.1	116.979	13.807(205)	15.681(202)	29.243	0.036
	1,000,000/1,369,977	0.2	104.356	11.401(186)	12.878(183)	25.752	0.130
	5,000,000/5,463,192	0.5	91.508	9.527(159)	10.617(157)	20.217	0.183
	10,000,000/10,729,824	0.9	86.061	9.824(147)	10.758(145)	17.620	0.169
1	1/1	0.0	677.397^c^	–93.025^d^	124.713	33.145^e^	–0.047^f^
	50,000/50,010	0.0	300.824	35.294(413)	50.017(390)	32.563	–0.044
	100,000/100,035	0.0	237.746	31.657(406)	39.836(390)	32.398	–0.027
	250,000/400,180	0.1	142.976	26.006(234)	28.422(229)	30.664	–0.017
	500,000/850,568	0.2	120.112	23.424(190)	25.046(187)	27.514	0.056
	1,000,000/1,600,766	0.2	111.708	21.860(176)	23.221(173)	24.619	0.138
	5,000,000/6,403,314	0.6	100.023	19.912(159)	20.942(157)	19.293	0.178
	10,000,000/12,806,196	1.0	94.485	18.841(151)	19.737(149)	16.670	0.162

a For each value of λ, the *E*_var_, *E*_var_ + Δ*E* ^(2)^, and *E*_var_ + Δ*E*_r_^(2)^ energies are reported as errors, in millihartree, relative to the extrapolated *E*_var_ + Δ*E*_r_^(2)^ energy found using a linear fit based on the last four *E*_var,*k*_ + Δ*E*_r,*k*_^(2)^ values leading to the largest CIPSI wave function obtained with *N*_det(in)_ = 10,000,000, plotted against the corresponding Δ*E*_r,*k*_^(2)^ corrections, following the procedure described in refs 41, 78, and 163 . The extrapolated *E*_var_ + Δ*E*_r_^(2)^ energies at λ = 0 and 1 are −154.331596(4818) and −154.373574(1906) hartree, respectively, where the error bounds in parentheses correspond to the uncertainty associated with the linear fit. The error bounds for the *E*_var_ + Δ*E* ^(2)^ and *E*_var_ + Δ*E*_r_^(2)^ energies obtained at the various values of *N*_det(in)_ reflect on the semistochastic design of the $V_{\mathrm{ext}}^{\left(k\right)}$ spaces discussed in the main text, but they ignore the uncertainties characterizing the reference *E*_var_ + Δ*E*_r_^(2)^ energies obtained in the above extrapolation procedure.

b The CC­(*P*) and CC­(*P*;*Q*) energies are reported as errors relative to CCSDT, in millihartree. The total CCSDT energies at λ = 0 and 1 are −154.339738 and −154.370744 hartree, respectively.

c Equivalent to ROHF.

d Equivalent to the result obtained with the second-order MBPT approach using the Epstein–Nesbet denominator.

e Equivalent to CCSD.

f Equivalent to CR-CC­(2,3).

**Table 6:** Convergence of the CC­(*P*) and CC­(*P*;*Q*) Singlet–Triplet Gaps Δ*E*_S–T_ = *E*_S_ – *E*_T_ Characterizing Cyclobutadiene, as Described by the cc-pVTZ Basis Set, Toward Their CCSDT Parents at the R (λ = 0) and TS (λ = 1) Geometries, Along with the Δ*E*_S–T_ Data Resulting from the Associated Variational and Perturbatively Corrected CIPSI Computations

λ	*N*_det(in)_/*N*_det(out)_	% of triples	*E*_var_ ^a^	*E*_var_ + Δ*E* ^(2)^ ^a^	*E*_var_ + Δ*E*_r_^(2)^ ^a^	CC(*P*)^b^	CC(*P*;*Q*)^b^
0	1/1; 1	0.0; 0.0	20.797^c^	19.764^d^	20.275	1.295^e^	0.605^f^
	50,000/88,980; 85,613	0.0; 0.0	–27.586	10.714(294)	7.480(283)	1.790	0.593
	100,000/177,965; 167,642	0.0; 0.0	–18.412	10.560(260)	8.888(253)	1.721	0.549
	250,000/355,932; 341,366	0.0; 0.1	–3.050	11.162(203)	10.552(199)	0.773	0.403
	500,000/711,877; 684,969	0.0; 0.1	7.978	11.202(177)	11.062(174)	0.007	0.253
	1,000,000/1,423,810; 1,369,977	0.1; 0.2	11.250	10.958(165)	10.946(162)	0.227	0.144
	5,000,000/5,695,067; 5,463,192	0.5; 0.5	10.619	10.028(144)	10.030(142)	–0.074	0.068
	10,000,000/11,390,227; 10,729,824	0.9; 0.9	9.604	8.970(134)	8.962(132)	–0.454	0.042
1	1/1; 1	0.0; 0.0	41.644^c^	5.155^d^	126.403	13.843^e^	8.685^f^
	50,000/83,877; 50,010	0.0; 0.0	–38.625	20.559(322)	14.758(307)	13.397	7.619
	100,000/169,536; 100,035	0.0; 0.0	–25.800	20.710(303)	17.571(292)	12.338	6.866
	250,000/339,078; 400,180	0.0; 0.1	18.815	21.880(195)	21.778(191)	9.559	4.482
	500,000/678,157; 850,568	0.0; 0.2	23.944	18.643(178)	18.834(175)	5.282	1.406
	1,000,000/1,342,908; 1,600,766	0.1; 0.2	17.555	10.819(168)	11.028(165)	0.891	0.285
	5,000,000/5,425,949; 6,403,314	0.3; 0.6	10.255	8.066(145)	8.121(143)	–0.425	0.212
	10,000,000/10,744,113; 12,806,196	0.6; 1.0	9.671	7.776(137)	7.819(135)	–0.309	0.161

a For each value of λ, the *E*_var_, *E*_var_ + Δ*E* ^(2)^, and *E*_var_ + Δ*E*_r_^(2)^ singlet–triplet gaps are reported as errors, in kcal/mol, relative to the parent CIPSI data obtained by forming the differences between the extrapolated *E*_var_ + Δ*E*_r_^(2)^ energies of the lowest singlet and triplet states given in footnotes “a” of Tables 4 and 5. The resulting reference *E*_var_ + Δ*E*_r_^(2)^ singlet–triplet gap values at λ = 0 and 1 are −41.208(3.254) and −9.593(2.111) kcal/mol, respectively.

b The CC­(*P*) and CC­(*P*;*Q*) singlet–triplet gaps are reported as errors relative to CCSDT, in kcal/mol. The CCSDT singlet–triplet gap values at λ = 0 and 1 are −32.019 and −1.981 kcal/mol, respectively.

c Equivalent to RHF/ROHF.

d Equivalent to the result obtained with the second-order MBPT approach using the Epstein–Nesbet denominator.

e Equivalent to CCSD.

f Equivalent to CR-CC­(2,3).

We conclude this section by noticing that the results reported in Tables 1–3 for the cc-pVDZ basis and Tables 4–6 for cc-pVTZ also show that the convergence of the CIPSI-driven CC­(*P*) and CC­(*P*;*Q*) energies of the lowest ^1^ *A*_*g*_(*D*_2h_) and ^3^ *B*_1*g*_(*D*_2h_) states of cyclobutadiene and the gap between them toward their respective CCSDT parents with *N*_det(in)_ or *N*_det(out)_ is faster than that characterizing the associated variational and perturbatively corrected CIPSI energetics toward the extrapolated *E*_var_ + Δ*E*_r_^(2)^ values (this is particularly true for the larger cc-pVTZ basis set, where we would have to consider much larger *N*_det(in)_ values and diagonalization spaces in CIPSI than those used in this study to obtain more accurate estimates of the extrapolated *E*_var_ + Δ*E*_r_^(2)^ energies). This observation is consistent with our initial study announcing the CIPSI-based CC­(*P*) and CC­(*P*;*Q*) methodologies^125^ and the fact that the CC­(*P*;*Q*) calculations are capable of accurately approximating the parent CCSDT energetics out of the unconverged CIPSI runs using relatively small Hamiltonian diagonalization spaces, even when *T*_3_ correlations are large and nonperturbative and electronic quasi-degeneracies become substantial, as is the case in the barrier region of the ground-state ^1^ *A*_*g*_(*D*_2h_) potential. While this may not be a general remark, we also observe that the perturbatively corrected *E*_var_ + Δ*E* ^(2)^ and *E*_var_ + Δ*E*_r_^(2)^ energies of the lowest singlet and triplet states of cyclobutadiene and the gap between them converge toward their extrapolated limits at a rate similar to that characterizing our uncorrected CC­(*P*) calculations toward CCSDT. The δ­(*P*;*Q*)-corrected CC­(*P*;*Q*) energetics converge to CCSDT much faster. This might be yet another way of looking at the effectiveness of δ­(*P*;*Q*) moment corrections in improving the underlying CC­(*P*) results. That being said, we should keep in mind that the algorithms used to obtain the CCSDT and the perturbatively corrected and extrapolated CIPSI energies are fundamentally different procedures. Furthermore, and more importantly given the objectives of this study, where we are interested in exploring the CIPSI-driven CC­(*P*) and CC­(*P*;*Q*) methodologies, not the CIPSI approach itself, the CIPSI wave function growth in the calculations reported in Tables 1–6 (especially in Tables 4–6 for the cc-pVTZ basis) was terminated long before our CIPSI runs were well converged, as we only needed information about the leading triply excited determinants and were not interested in saturating the triply excited manifolds of the relevant many-electron Hilbert spaces. Last but not least, to highlight the robustness of our CC­(*P*;*Q*) framework, all of the calculations reported in this work relied on the RHF and ROHF orbitals, i.e., we made no attempt to further optimize orbitals to make them consistent with correlated computations, which would improve CIPSI’s performance and which might also help our CC­(*P*) and CC­(*P*;*Q*) results using smaller *N*_det(in)_ values, especially in the vicinity of the square TS geometry. We intend to look into the potential benefits that might be offered by orbital optimizations in the CIPSI-driven CC­(*P*) and CC­(*P*;*Q*) calculations in a future study.

## 4 Summary and Concluding Remarks

An accurate determination of singlet–triplet gaps in biradicals represents a formidable test for *ab initio* electronic structure methodologies, as it requires balancing strong nondynamical many-electron correlation effects, needed for a reliable description of the low-spin singlet states that have a manifestly multiconfigurational nature, with the generally weaker, largely dynamical, correlations characterizing the high-spin triplet states. Although high-level CC methods with a full treatment of higher-than-two-body clusters, such as CCSDT or CCSDTQ, are often powerful enough to capture the dynamical and nondynamical correlation effects relevant in such studies, their applications are hindered by the demanding computational steps and memory requirements, which are prohibitively expensive when larger many-electron systems are examined. One of the promising ideas aimed at addressing this situation within the single-reference CC framework is the CC­(*P*;*Q*) formalism, in which one solves the CC amplitude equations in a suitably defined subspace of the many-electron Hilbert space, referred to as the *P* space, and improves the resulting CC­(*P*) energies using the *a posteriori* moment corrections, designated as δ­(*P*;*Q*), calculated with the help of the complementary *Q* space.^22,32,105,109,115,116,121–127^ Among the most attractive features of the CC­(*P*;*Q*) methodology is its flexibility, so that in addition to conventional choices of the *P* and *Q* spaces using truncations based on excitation ranks, which in the past resulted in the development of the biorthogonal CR-CC methods, such as the CR-CC­(2,3) triples correction to CCSD,^17,86–88^ one can consider various unconventional ways of setting up these spaces that can improve the CR-CC­(2,3), CCSD­(T), and similar energetics for systems with substantial electronic quasi-degeneracies by relaxing the *T*_1_ and *T*_2_ components of the cluster operator *T* in the presence of their higher-rank *T*_*n*_ counterparts with *n* > 2, such as *T*_3_, which become large, nonperturbative, and strongly coupled to *T*_1_ and *T*_2_ in such situations. This can be done without major increases in the computational effort by incorporating the leading higher-than-doubly excited determinants in the *P* spaces and using corrections δ­(*P*;*Q*) to capture the remaining correlations of interest.

In this work, we have examined the hybrid variant of the CC­(*P*;*Q*) methodology introduced in ref 125, in which the leading higher-than-doubly excited determinants in the *P* space are identified, in an automated fashion, using the sequences of Hamiltonian diagonalizations generated with the CIPSI algorithm.^76–78^ In order to thoroughly test the CIPSI-driven CC­(*P*;*Q*) formalism and obtain useful insights into its performance, we have focused on recovering the lowest-energy singlet and triplet potentials of cyclobutadiene along its automerization coordinate and the gap between them resulting from the full CCSDT computations which, based on comparisons with the perturbatively corrected and extrapolated CIPSI and DEA-EOMCC­(4p-2h)-level data, obtained in this study as well, provide reliable information. To do so, we have constructed an approximate, *D*_2h_-symmetric, one-dimensional automerization pathway connecting the rectangular reactant and product species via the square TS structure on the ground-state singlet potential using the information taken from ref 40 and performed a large number of CC­(*P*;*Q*) calculations for the lowest ^1^ *A*_*g*_(*D*_2h_) and ^3^ *B*_1*g*_(*D*_2h_) states of cyclobutadiene at the selected nuclear geometries along the resulting path, where for each state and for each geometry, we have explored a wide range of values of the CIPSI wave function termination parameter *N*_det(in)_ that controls the Hamiltonian diagonalization sequences preceding the CC­(*P*) and CC­(*P*;*Q*) runs. We have demonstrated that the CIPSI-driven CC­(*P*;*Q*) calculations are capable of accurately approximating the high-level CCSDT energetics of the lowest singlet and triplet states of cyclobutadiene across the entire automerization pathway, to within small fractions of a millihartree for total energies and 0.1–0.3 kcal/mol for the singlet–triplet gaps, using tiny fractions of the triply excited determinants, on the order of 1% of all triples for the cc-pVDZ basis set and 0.1–0.2% for cc-pVTZ, in the underlying *P* spaces extracted from the relatively inexpensive CIPSI diagonalizations in spaces that are orders of magnitude smaller than the numbers of cluster amplitudes used by CCSDT. This extraordinary performance of the CIPSI-driven CC­(*P*;*Q*) approach applies to both the less demanding reactant/product region, where the lowest singlet and triplet states of cyclobutadiene are largely single-configurational and the absolute values of the singlet–triplet gap exceed 30 kcal/mol, and the vicinity of the TS structure on the ground-state singlet potential, where the high-spin triplet state retains its weakly correlated, single-determinantal, nature, but the singlet state, separated from its triplet counterpart by only a few kcal/mol, becomes multiconfigurational, strongly correlated, and characterized by large and highly nonperturbative *T*_3_ correlations, which are strongly coupled to the one- and two-body components of *T* and which result in failure of CR-CC­(2,3) and other noniterative triples corrections to CCSD. Interestingly, the uncorrected CC­(*P*) computations using similarly compact excitation spaces can be accurate as well, reproducing the CCSDT values of the singlet–triplet gap across the entire automerization pathway to within ∼1–2 kcal/mol, improving the poor CCSD and CR-CC­(2,3) results in the vicinity of the TS geometry, and reaffirming the usefulness of incorporating the leading triply excited determinants into the underlying *P* spaces, but the total energies of the lowest ^1^ *A*_*g*_(*D*_2h_) and ^3^ *B*_1*g*_(*D*_2h_) states of cyclobutadiene obtained with the CC­(*P*) approach converge to their CCSDT parents with *N*_det(in)_ very slowly, so one has to rely on error cancellations to obtain accurate singlet–triplet gaps with CC­(*P*). The δ­(*P*;*Q*) corrections are very helpful in this regard. They reduce errors in the total CC­(*P*) energies of the ^1^ *A*_*g*_(*D*_2h_) and ^3^ *B*_1*g*_(*D*_2h_) states of cyclobutadiene by orders of magnitude while substantially improving the resulting singlet–triplet gaps, making their convergence toward CCSDT smoother and more systematic. Given the relatively low costs of determining the δ­(*P*;*Q*) corrections compared to the preceding CIPSI and CC­(*P*) steps and the enormous benefits resulting from their application in the CIPSI-driven CC­(*P*;*Q*) calculations, we recommend using CC­(*P*;*Q*).

The excellent performance of the CIPSI-driven CC­(*P*;*Q*) approach in converging the lowest-energy singlet and triplet potentials of cyclobutadiene obtained with CCSDT, observed in this study, along with the promising initial results reported in ref 125, motivate us to pursue the hybrid CC methodologies combining the CC­(*P*;*Q*) framework with selected CI even further. We will, for example, investigate how much the CC­(*P*;*Q*) singlet and triplet potentials reported in this work, especially the ground-state singlet potential in the vicinity of the TS geometry, can benefit from replacing the RHF and ROHF orbitals exploited in the calculations reported in this work by the suitably optimized orbitals consistent with the CC­(*P*) or CIPSI wave functions. We will also examine how much each of the singlet and triplet potentials of cyclobutadiene obtained with the CIPSI-driven CC­(*P*;*Q*) approach using a given value of *N*_det(in)_, especially its smoothness, can improve by consolidating the *P* spaces corresponding to the different geometries along the automerization path or, to be more precise in the context of the CC­(*P*;*Q*) calculations aimed at recovering the CCSDT energetics performed in this study, by merging the triple excitation manifolds incorporated in those spaces. Among other topics worth exploring, it will be interesting to examine if anything substantial can be gained by replacing the CIPSI algorithm in our CC­(*P*;*Q*) considerations by other selected CI techniques, such as heat-bath CI,^156–158^ adaptive CI,^150,151^ or adaptive sampling CI.^152,153^ Last but not least, following the strategy adopted in our previous work on the semistochastic, CIQMC-driven, CC­(*P*;*Q*) approaches,^122–124^ we are planning to extend the CIPSI-driven CC­(*P*;*Q*) methodology investigated in this study and ref 125 to higher CC levels, especially CCSDTQ, and excited electronic states, with an initial focus on converging the EOMCCSDT^165–167^ energetics, while seeking additional savings in the computational effort by replacing the unconstrained CIPSI algorithm, which is allowed to explore the entire many-electron Hilbert space, by its truncated analogs consistent with the determinantal spaces needed in the target CC calculations (e.g., the CISDT or CISDTQ analogs of CIPSI when attempting to use the CIPSI-driven CC­(*P*;*Q*) framework to converge the CCSDT or CCSDTQ energetics).

## Appendix: Key Elements of the Algorithm Used to Implement the CC(*P*) Amplitude Equations, Along with Illustrative Timings

As pointed out in Section 2, the key to achieving computational efficiency in the CC­(*P*;*Q*) calculations, including the CIPSI-driven CC­(*P*;*Q*) approach aimed at converging the CCSDT energetics examined in this study, lies in the development of an algorithm capable of offering substantial speedups compared to the parent CC method when the lists of higher-than-doubly excited determinants included in the *P* spaces used in the CC­(*P*) iterations do not necessarily form continuous manifolds. The most essential ingredients of our strategy for implementing the CC­(*P*) amplitude equations, eq 4, and the companion left-eigenstate system, eq 10, in which the *P* space used to define the cluster operator *T* ^(*P*)^ and its deexcitation Λ^(*P*)^ counterpart consists of all singly and doubly excited determinants, |Φ_*i*_^*a*^⟩ and |Φ_*ij*_^*ab*^⟩, respectively, and a potentially spotty subset of triply excited determinants |Φ_*ijk*_^*abc*^⟩, identified in this work by CIPSI, are summarized in this appendix. In the interest of space, in the description below, we focus on the CC­(*P*) amplitude equations that are used to determine the cluster operator

A1 $$T^{\left(P\right)}=T_{1}+T_{2}+T_{3}^{\left(P\right)},$$

where, consistent with the above definition of the *P* space, designated as $H^{\left(P\right)}$ , the one- and two-body components of *T* ^(*P*)^,

A2 $$T_{1}=\underset{i,a}{\sum }t_{a}^{i}E_{i}^{a}$$

and

A3 $$T_{2}=\underset{i<j,a<b}{\sum }t_{ab}^{ij}E_{ij}^{ab},$$

respectively, are treated fully, but the three-body component

A4 $$T_{3}^{\left(P\right)}=\underset{\left|\Phi_{ijk}^{abc}\right\rangle \in H^{\left(P\right)}}{\sum }t_{abc}^{ijk}E_{ijk}^{abc}$$

is defined on a subset of triply excited determinants that do not have to form a continuous manifold (if the list of triples in *T*_3_^(*P*)^ is extracted from the terminal CIPSI wave function |Ψ^(CIPSI)^⟩, *T*_3_^(*P*)^ becomes the *T*_3_^(CIPSI)^ operator introduced in Section 2). Following the notation used in Section 2, *E*_*i*_^*a*^, *E*_*ij*_^*ab*^, and *E*_*ijk*_^*abc*^ in eqs 13–15 are the elementary particle–hole excitation operators that generate the |Φ_*i*_^*a*^⟩, |Φ_*ij*_^*ab*^⟩, and |Φ_*ijk*_^*abc*^⟩ determinants when acting on the reference function |Φ⟩ and *t*_*a*_^*i*^, *t*_*ab*_^*ij*^, and *t*_*abc*_^*ijk*^ are the cluster amplitudes defining *T*_1_, *T*_2_, and *T*_3_^(*P*)^, respectively. Our implementation of the left-eigenstate CC­(*P*) system, needed to obtain the deexcitation operator Λ^(*P*)^ = Λ_1_ + Λ_2_ + Λ_3_^(*P*)^, in which the triples entering Λ_3_^(*P*)^ are the same as those included in *T*_3_^(*P*)^, uses the same philosophy as that adopted in handling the CC­(*P*) amplitude equations, so we are not discussing it here. A more complete description of our algorithm used to efficiently handle the right and left CC­(*P*) equations, eqs 4 and 10, assuming the above definitions of *T* ^(*P*)^, Λ^(*P*)^, and the underlying *P* space, and of CCpy, in which the CIPSI-driven, adaptive, and active-orbital-based CC­(*P*;*Q*) approaches targeting CCSDT have been implemented, will be discussed in a separate publication.

To formulate our algorithm for the CC­(*P*) amplitude equations that leads to the desired speedups compared to the parent CCSDT approach, we first isolate the contributions due to the three-body component *T*_3_^(*P*)^ of the cluster operator *T* ^(*P*)^ by expanding eq 4, in which *T* ^(*P*)^ is defined by eq 12, as follows:

A5 $$M_{K}\left(2\right)+\left\langle \Phi_{K}\right|\left[{\mathrm{H̅}}^{\left(2\right)},T_{3}^{\left(P\right)}\right]\left|\Phi \right\rangle =0,\;\left|\Phi_{K}\right\rangle \in H^{\left(P\right)}.$$

The |Φ_*K*_⟩s in eq 16 are the singly, doubly, and selected triply excited determinants included in the *P* space,

A6 $${\mathrm{H̅}}^{\left(2\right)}=e^{-T_{1}-T_{2}}He^{T_{1}+T_{2}}$$

is the Hamiltonian transformed with the *e* ^*T*_1_+*T*_2_^ part of *e* ^*T* ^(*P*)^^, and

A7 $$M_{K}\left(2\right)=\left\langle \Phi_{K}\right|{\mathrm{H̅}}^{\left(2\right)}\left|\Phi \right\rangle$$

are the quantities resembling the generalized moments of the CCSD equations, except that the *T*_1_ and *T*_2_ amplitudes used to construct them originate from the CC­(*P*) iterations, i.e., they are relaxed in the presence of the *T*_3_^(*P*)^ contribution to *T* ^(*P*)^. It should be noted that with the definitions of $H^{\left(P\right)}$ and *T* ^(*P*)^ considered here, terms nonlinear in *T*_3_^(*P*)^ do not contribute to the CC­(*P*) amplitude equations. Thus, after straightforward manipulations following the insertion of eq 15 for *T*_3_^(*P*)^ into the commutator (or the equivalent connected product of *H̅* ^(2)^ and *T*_3_^(*P*)^) appearing in eq 16, we obtain

A8 $$\underset{\left(I\right)}{\underset{︸}{M_{K}\left(2\right)\;}}+\underset{\left(\mathrm{II}\right)}{\underset{︸}{\underset{\left|\Phi_{lmn}^{def}\right\rangle \in H^{\left(P\right)}}{\sum }\left\langle \Phi_{K}\right|{\mathrm{H̅}}_{N}^{\left(2\right)}\left|\Phi_{lmn}^{def}\right\rangle t_{def}^{lmn}}}=0,\;\left|\Phi_{K}\right\rangle \in H^{\left(P\right)},$$

where *H̅*_*N*_^(2)^ = *H̅* ^(2)^ − *E* ^(*P*)^ **1** is the *H̅* ^(2)^ operator in the normal-product form with respect to the Fermi vacuum |Φ⟩, with *E* ^(*P*)^ representing the ground-state CC­(*P*) energy, eq 6, and **1** designating the unit operator (because of the absence of higher-than-two-body interactions in the Hamiltonians used in quantum chemistry, the *T*_3_^(*P*)^ contribution to *T* ^(*P*)^ does not enter the formula for *E* ^(*P*)^, i.e., *E* ^(*P*)^ = ⟨Φ|*H̅* ^(2)^|Φ⟩).

Equation 19 represents the core equation underlying our CC­(*P*) algorithm. The programmable expressions for the matrix elements defining the one- and two-body components of *H̅*_*N*_^(2)^, denoted as *h̅*_*p*_^*q*^ and *h̅*_*pq*_^*rs*^, respectively, in terms of the one- and two-body cluster amplitudes *t*_*a*_^*i*^ and *t*_*ab*_^*ij*^ and one- and two-electron integrals in a molecular spin-orbital basis, designated as *f*_*p*_^*q*^ = ⟨*p*|*f*|*q*⟩, where *f* is the Fock operator, and *v*_*pq*_^*rs*^ = ⟨*pq*|*v*|*rs*⟩ − ⟨*pq*|*v*|*sr*⟩, where *v* is the electron–electron interaction, which define the Hamiltonian in the normal-ordered form, *H*_*N*_ = *H* – ⟨Φ|*H*|Φ⟩, in a usual manner, are well known and can be found, for example, in Table I of ref 111 (indices *p*, *q*, *r*, *s* refer to generic—meaning occupied as well as unoccupied—molecular spin-orbitals). In each iteration of the CC­(*P*) procedure, the *h̅*_*p*_^*q*^ and *h̅*_*pq*_^*rs*^ matrix elements are recalculated with the current values of *T*_1_ and *T*_2_ and stored as computational intermediates, which allows us to evaluate eq 19 in a factorized form. In our current implementation, the CC­(*P*) system is constructed as the sum of two distinct contributions, referred to in eq 19 as terms (I) and (II). Each of these two terms is computed in an efficient fashion using a dedicated strategy tailored to its structure, which we discuss next.

### Evaluation of Term (I) in Equation A8

As already alluded to above, the first term in eq 19 resembles the generalized moments of the CCSD equations. In particular, when |Φ_*K*_⟩ = |Φ_*i*_^*a*^⟩ or |Φ_*ij*_^*ab*^⟩, the resulting one- and two-body moments,

A9 Mai(2)=⟨Φia|H̅(2)|Φ⟩≡h̅a i

and

A10 Mabij(2)=⟨Φijab|H̅(2)|Φ⟩≡h̅ab ij,

respectively, are equivalent to the left-hand sides of the standard CCSD amplitude equations, in which the singly and doubly excited cluster amplitudes [that in CCSD originate from setting $M_{a}^{i}\left(2\right)$ and $M_{ab}^{ij}\left(2\right)$ to zero] are obtained in the process of solving the CC­(*P*) equations, eq 16 or 19, for the cluster operator *T* ^(*P*)^ defined by eq 12. As is well established, the $M_{a}^{i}\left(2\right)$ and $M_{ab}^{ij}\left(2\right)$ expressions can be computed in a fully vectorized fashion (i.e., avoiding the use of explicit loops) by taking advantage of efficient matrix multiplication and transposition routines provided by BLAS. When the *P* space $H^{\left(P\right)}$ contains all singly and doubly excited determinants, which is the case in the CIPSI-driven CC­(*P*;*Q*) calculations aimed at converging the CCSDT energetics, such as those discussed in the present article, the evaluation of $M_{a}^{i}\left(2\right)$ and $M_{ab}^{ij}\left(2\right)$ involves the usual $n_{o}^{2}n_{u}^{4}$ and other, less expensive, computational steps characterizing CCSD, where *n*_*o*_ (*n*_*u*_) is the number of correlated occupied (unoccupied) spin-orbitals in |Φ⟩.

With the exception of the source of *T*_1_ and *T*_2_, which in the CC­(*P*) calculations originate from solving the system given by eq 16 or 19, and besides the fact that the projections on the triply excited determinants |Φ_*ijk*_^*abc*^⟩ in eq 18 entering eq 19 are limited to the |Φ_*ijk*_^*abc*^⟩s included in the *P* space $H^{\left(P\right)}$ , the programmable expressions for the three-body moments

A11 $$M_{abc}^{ijk}\left(2\right)=\left\langle \Phi_{ijk}^{abc}\right|{\mathrm{H̅}}^{\left(2\right)}|\Phi \rangle ,$$

in terms of the singly and doubly cluster amplitudes and one- and two-body matrix elements of *H̅*_*N*_^(2)^, are identical to those exploited in methods such as CR-CC­(2,3). Assuming the Einstein summation convention over repeated upper and lower indices used in the remainder of this appendix, they are (cf., e.g., eqs 59 and 62 in ref 111)­

A12 Mabcijk(2)=12Ai/jkAabc(h̅ab ietecjk−Iamijtbcmk),

where *I*_*am*_^*ij*^ = *h̅*_*am*_^*ij*^ − *h̅*_*m*_^*e*^ *t*_*ae*_^*ij*^ and $A_{p/qr}$ ≡ $A^{p/qr}$ = 1 − (*pq*) − (*pr*) and $A_{pqr}$ ≡ $A^{pqr}$ = 1 − (*pq*) − (*pr*) − (*qr*) + (*pqr*) + (*prq*) are index antisymmetrizers [with (*pq*) representing a transposition of *p* and *q*]. For each |Φ_*ijk*_^*abc*^⟩ ∈ $H^{\left(P\right)}$ , eq 27 is computed by forming dot products of *h̅*_*ab*_^*ie*^ with *t*_*ec*_^*jk*^ (summed over *e*) and *I*_*am*_^*ij*^ with *t*_*bc*_^*mk*^ (summed over *m*), which can be efficiently done with fast matrix multiplication routines from the BLAS library, and subtracting the latter from the former. Similar remarks apply to the (−*h̅*_*m*_^*e*^ *t*_*ae*_^*ij*^) term in the definition of the *I*_*am*_^*ij*^ intermediate (which is a dot product involving summation over *e*) and the fully vectorized expressions for the one- and two-body matrix elements of *H̅*_*N*_^(2)^ entering *I*_*am*_^*ij*^ and the right-hand side of eq 27, provided, for example, in Table I of ref 111. In the limit of the *P* space including all triply excited determinants (as in, e.g., full CCSDT), evaluation of eq 27 involves *n*_*o*_^3^ *n*_*u*_^4^ operations. However, when the *P* space contains only a small subset of triply excited determinants, as is the case in our CIPSI-driven CC­(*P*;*Q*) calculations, the cost of evaluating eq 27 is reduced relative to CCSDT by a factor of (*D*/*d*), where *D* is the number of all triply excited determinants |Φ_*ijk*_^*abc*^⟩ and *d* is the subset of those |Φ_*ijk*_^*abc*^⟩s that are included in the *P* space $H^{\left(P\right)}$ .

### Evaluation of Term (II) in Equation A8

We now turn our attention to term (II) in eq 19, which captures the contributions to the CC­(*P*) amplitude equations that are linear in *T*_3_^(*P*)^. A simple recipe for evaluating this term, which we have adopted in our CC­(*P*) algorithm implemented in CCpy, consists of computing matrix elements of *H̅*_*N*_^(2)^ in the singles–triples (ST) sector, ⟨Φ_*i*_^*a*^|*H̅*_*N*_^(2)^|Φ_*lmn*_^*def*^⟩, doubles–triples (DT) sector, ⟨Φ_*ij*_^*ab*^|*H̅*_*N*_^(2)^|Φ_*lmn*_^*def*^⟩, and triples–triples (TT) sector, ⟨Φ_*ijk*_^*abc*^|*H̅*_*N*_^(2)^|Φ_*lmn*_^*def*^⟩, where the triply excited determinants |Φ_*ijk*_^*abc*^⟩ and |Φ_*lmn*_^*def*^⟩ are limited to those included in the *P* space, and multiplying the resulting ST, DT, and TT blocks of the matrix representing *H̅*_*N*_^(2)^ with the vector of three-body cluster amplitudes corresponding to |Φ_*lmn*_^*def*^⟩s belonging to $H^{\left(P\right)}$ using a standard definition of the matrix–vector product. The programmable expressions for the ST, DT, and TT blocks of the matrix representing *H̅*_*N*_^(2)^ exploited in our work, focusing on the contributions that can be written in terms of the one- and two-body matrix elements *h̅*_*p*_^*q*^ and *h̅*_*pq*_^*rs*^ (which can be efficiently determined using the vectorized expressions provided in Table I of ref 111), i.e., excluding terms in the TT block that involve the three-body component of the *H̅*_*N*_^(2)^ operator, which, as further elaborated on below, may require a different treatment, are shown in Table 7.

In our present implementation of eq 19, we evaluate term (II) using two nested loops, where the outer loop runs over the bra states ⟨Φ_*K*_| that correspond to the projections on all singly and doubly excited determinants, |Φ_*i*_^*a*^⟩ and |Φ_*ij*_^*ab*^⟩, respectively, and the subset of triply excited determinants |Φ_*ijk*_^*abc*^⟩ included in the *P* space, and an inner loop enumerates the ket states associated with the triply excited determinants |Φ_*lmn*_^*def*^⟩ ∈ $H^{\left(P\right)}$ matching the content of *T*_3_^(*P*)^. For all pairs of the bra (⟨Φ_*K*_|) and ket (|Φ_*lmn*_^*def*^⟩) determinants specified within these two loops, the corresponding matrix elements ⟨Φ_*K*_|*H̅*_*N*_^(2)^|Φ_*lmn*_^*def*^⟩ can be evaluated using the formulas provided in Table 7, which are subsequently multiplied by the triply excited cluster amplitudes *t*_*def*_^*lmn*^ defining *T*_3_^(*P*)^ associated with |Φ_*lmn*_^*def*^⟩ ∈ $H^{\left(P\right)}$ . It should be noted though that in addition to the terms that originate from the one- and two-body components of the *H̅*_*N*_^(2)^ operator considered in Table 7, the TT block of the matrix representing *H̅*_*N*_^(2)^ also contains contributions that engage its three-body component arising from ⟨Φ_*ijk*_^*abc*^|​[[*H*_*N*_,*T*_2_],*T*_3_^(*P*)^]|​Φ⟩, which are not accounted for in Table 7. One could determine these contributions within the two nested loops over ⟨Φ_*K*_| and |Φ_*lmn*_^*def*^⟩ discussed here (after augmenting Table 7 with the expressions due to the three-body component of *H̅*_*N*_^(2)^), but it is much more efficient, in terms of CPU time and storage costs, to determine them during the previously described evaluation of moments $M_{abc}^{ijk}\left(2\right)$ that enter term (I) of eq 19. This can be accomplished by rewriting the contributions to ⟨Φ_*ijk*_^*abc*^​|​[[*H*_*N*_,*T*_2_],​*T*_3_^(*P*)^]|​Φ⟩ that originate from the three-body component of *H̅*_*N*_^(2)^, such that the Hamiltonian is first contracted with *T*_3_^(*P*)^ rather than *T*_2_, and combining the resulting expression with the three-body moment $M_{abc}^{ijk}\left(2\right)$ . In practice, all one has to do is to dress *h̅*_*ab*_^*ie*^ and *I*_*am*_^*ij*^ in eq 27 with the suitably defined *T*_3_^(*P*)^-dependent terms and compute

A13 $${\mathrm{M̃}}_{abc}^{ijk}\left(2\right)=\frac{1}{2}A^{i/jk}A_{abc}({\mathrm{h̃}}_{ab}^{ie}t_{ec}^{jk}-{Ĩ}_{am}^{ij}t_{bc}^{mk}),$$

where ${\mathrm{h̃}}_{ab}^{ie}$ = h̅ab ie − 12h̅mn eftabfimn ≡ h̅ab ie − $\frac{1}{2}v_{mn}^{ef}t_{abf}^{imn}$ and ${Ĩ}_{am}^{ij}$ = $I_{am}^{ij}$ + 12h̅mn eftaefijn ≡ $I_{am}^{ij}$ + $\frac{1}{2}v_{mn}^{ef}t_{aef}^{ijn}$ , instead of the original moments $M_{abc}^{ijk}\left(2\right)$ given by eq 27. In this way, we can generate the contributions to the ⟨Φ_*ijk*_^*abc*^|​[[*H*_*N*_,*T*_2_],*T*_3_^(*P*)^]|​Φ⟩ term due to the three-body component of *H̅*_*N*_^(2)^, which would otherwise have to be incorporated in the TT block of the matrix representing *H̅*_*N*_^(2)^, outside the two loops over ⟨Φ_*K*_| and |Φ_*lmn*_^*def*^⟩ from $H^{\left(P\right)}$ used to treat the rest of term (II) and without having to calculate and store the memory-demanding six-index matrix elements associated with the second-quantized formula for the three-body component of the *H̅*_*N*_^(2)^ operator. To keep the costs of our computations as low as possible, the matrix multiplications involving the summations over *m*, *n*, and *f* in the (−12h̅mn eftabfimn) contribution to *h̃*_*ab*_^*ie*^ and *n*, *e*, and *f* in the 12h̅mn eftaefijn contribution to *Ĩ*_*am*_^*ij*^ are executed by looping over the triply excited amplitudes seen in these expressions, which span the subset of triples included in *T*_3_^(*P*)^. The determination of the *T*_3_^(*P*)^ contributions to the CC­(*P*) amplitude equations represented in eq 19 by term (II) via the matrix−vector products involving the ST, DT, and TT blocks of the matrix representing *H̅*_*N*_^(2)^ is reminiscent of the strategies adopted in selected CI codes. It allows us to accommodate arbitrary or irregular lists of triply excited determinants included in the *P* space and defining the *T*_3_^(*P*)^ cluster operator, which may not form continuous excitation manifolds labeled by occupied and unoccupied orbitals from the respective ranges of indices. As illustrated by the computational timings provided at the end of this appendix, the benefits of implementing the CC­(*P*) approach in this manner become enormous when the fraction of triples included in the *P* space is very small, which is the main point of all CC­(*P*;*Q*) methods that we have pursued so far, including the CIPSI-driven variant examined in this study.

In order to reduce the computational effort involved in determining term (II) in eq 19 using the algorithm discussed in the preceding two paragraphs even further, in our implementation of the CC­(*P*) approach in CCpy, we also take advantage of the sparsity of the matrix representing *H̅*_*N*_^(2)^ in the subspace of the many-electron Hilbert space spanned by singly, doubly, and triply excited determinants. In particular, close inspection of the Kronecker deltas entering the formulas for the matrix elements of the *H̅*_*N*_^(2)^ operator listed in Table 7 shows that ⟨Φ_*K*_|*H̅*_*N*_^(2)^|Φ_*lmn*_^*def*^⟩ is zero unless the bra and ket determinants in it share some or all of their hole and particle indices. In analogy to the well-known 0-, 1-, and 2-electron Slater rules used to evaluate the nonzero matrix elements of the bare electronic Hamiltonian, the nonzero matrix elements of the similarity-transformed Hamiltonian *H̅*_*N*_^(2)^ in the ST, DT, and TT sectors entering term (II) of eq 19 can be classified according to the numbers of differences occurring in the hole and particle indices characterizing ⟨Φ_*K*_| and |Φ_*lmn*_^*def*^⟩. Thus, in our CC­(*P*) algorithm, we adopt the language in which if the bra and ket determinants entering ⟨Φ_*K*_|*H̅*_*N*_^(2)^|Φ_*lmn*_^*def*^⟩ differ in μ of their particle indices and *ν* of their hole indices, we call such ⟨Φ_*K*_|*H̅*_*N*_^(2)^|Φ_*lmn*_^*def*^⟩ a μ*p*-*νh*–difference matrix element. As shown in Table 7, each μ*p*-*νh*–difference matrix element ⟨Φ_*K*_|*H̅*_*N*_^(2)^|Φ_*lmn*_^*def*^⟩ engages a specific type of *h̅*_*p*_^*q*^ or *h̅*_*pq*_^*rs*^ and satisfies a distinct condition on the hole and particle indices in its bra and ket determinants to produce a nonzero contribution. To be more specific, if we define the set of hole (particle) indices characterizing the bra determinant ⟨Φ_*K*_| as *I*_*h*_ (*I*_*p*_) and the corresponding set of hole (particle) indices in the ket determinant |Φ_*lmn*_^*def*^⟩ as *J*_*h*_ (*J*_*p*_), then the index constraint applicable to each nonzero μ*p*-*νh*–difference matrix element ⟨Φ_*K*_|*H̅*_*N*_^(2)^|Φ_*lmn*_^*def*^⟩ considered in Table 7 can be mathematically expressed using the numbers of elements in the set intersections *S*_*h*_ = *I*_*h*_ ∩ *J*_*h*_ and *S*_*p*_ = *I*_*p*_ ∩ *J*_*p*_, i.e., via the cardinal numbers of *S*_*h*_ and *S*_*p*_, designated in Table 7 as |*S*_*h*_| and |*S*_*p*_|, respectively. The restrictions on these cardinal numbers, which must be imposed in order to obtain nonzero values of the matrix elements ⟨Φ_*K*_|*H̅*_*N*_^(2)^|Φ_*lmn*_^*def*^⟩ listed in Table 7, allow us to efficiently organize our work, identify the nonzero matrix elements very fast, and minimize the CPU operations involved in the evaluation and processing of the ST, DT, and TT blocks of *H̅*_*N*_^(2)^ that enter term (II) of eq 19. An example illustrating this statement, especially how the restrictions on |*S*_*h*_| and |*S*_*p*_| help, is discussed next.

Consider the evaluation of 2*p*-0*h*–difference matrix elements in the ⟨Φ_*ijk*_^*abc*^|*H̅*_*N*_^(2)^|Φ_*lmn*_^*def*^⟩ category (belonging to the TT block of *H̅*_*N*_^(2)^), which provide the contributions corresponding to the most expensive diagram in the CCSDT amplitude equations that in full CCSDT scales as *n*_*o*_^3^ *n*_*u*_^5^. As shown in Table 7, matrix elements of this type are evaluated according to the formula

A14 Ac/abAf/deh̅ab deδnkδliδmjδcf=δnkδliδmj(h̅ab deδcf−h̅cb deδaf−h̅ac deδbf−h̅ab feδcd+h̅cb feδad+h̅ac feδbd−h̅ab dfδce+h̅cb dfδae+h̅ac dfδbe),

where δ_*p*_^*q*^ is the Kronecker delta [since all indices in eq 21 and other similar expressions in Table 7 are fixed, and to remain consistent with the Einstein summation convention adopted in this work, upper and lower indices in eq 21 and Table 7 have been arranged such that the indices appearing on the same line are not summed over]. For a given pair of triply excited determinants |Φ_*ijk*_^*abc*^⟩ and |Φ_*lmn*_^*def*^⟩ belonging to the *P* space, eq 21 will evaluate to zero unless |*S*_*h*_| = 3 and |*S*_*p*_| = 1, where *S*_*h*_ = {*i*,*j*,*k*} ∩ {*l*,*m*,*n*} and *S*_*p*_ = {*a*,*b*,*c*} ∩ {*d*,*e*,*f*}. Our algorithm takes advantage of these restrictions on the cardinal numbers |*S*_*h*_| and |*S*_*p*_| required to obtain nonzero values of 2*p*-0*h*–difference matrix elements ⟨Φ_*ijk*_^*abc*^|*H̅*_*N*_^(2)^|Φ_*lmn*_^*def*^⟩ by partitioning the list of triply excited determinants entering the *P* space into nonoverlapping “buckets”, where each bucket contains triply excited determinants that share the same |*S*_*h*_| (in this case, 3) hole indices and the same |*S*_*p*_| (in this case, 1) particle indices. For example, if we have 4 electrons in a system and all determinants |Φ_*ijk*_^*abc*^⟩ in which *i* = 1, *j* = 2, and *k* = 3 and *c* = 8 are in the *P* space (in listing *P*-space triples, we always assume that *i* < *j* < *k* and *a* < *b* < *c*), the three determinants |Φ_123_^568^⟩, |Φ_123_^578^⟩, and |Φ_123_^678^⟩ that share three hole indices *i*, *j*, and *k* and one particle index *c* form one of the buckets. When the list of the *P*-space triples is organized in this fashion—with each bucket having |*S*_*h*_| hole indices and |*S*_*p*_| particle indices in common—we can simply skip the evaluation of 2*p*-0*h*–difference matrix elements of the ⟨Φ_*ijk*_^*abc*^|*H̅*_*N*_^(2)^|Φ_*lmn*_^*def*^⟩ type in which the bra and ket determinants belong to different buckets, as these will automatically evaluate to zero. In other words, we only determine those 2*p*-0*h*–difference matrix elements ⟨Φ_*ijk*_^*abc*^|*H̅*_*N*_^(2)^|Φ_*lmn*_^*def*^⟩ in which the |Φ_*ijk*_^*abc*^⟩ and |Φ_*lmn*_^*def*^⟩ determinants belong to the same bucket, repeating this process for all the buckets into which the list of the *P*-space triples has been partitioned. We can similarly exploit the sparsity patterns characterizing other μ*p*-*νh*–difference matrix elements of *H̅*_*N*_^(2)^ in the ST, DT, and TT categories listed in Table 7 by judiciously organizing the triply excited determinants included in the *P* space into the appropriately defined buckets based on common hole/particle indices, as indicated by the relevant values of |*S*_*h*_| and |*S*_*p*_|. In this way, we only have to execute the minimum number of CPU operations needed to evaluate nonzero contributions to term (II) in eq 19, which is the key to realizing the immense computational speedups offered by the short (but not necessarily regular) lists of triply excited determinants included in the *P* space. A detailed description of the numerical procedures used to partition the *P* space into the buckets of triply excited determinants relevant to the various types of ⟨Φ_*K*_|*H̅*_*N*_^(2)^|Φ_*lmn*_^*def*^⟩ matrix elements and μ*p*-*νh*–difference cases listed in Table 7, along with the associated spin-orbital index manipulations, will be presented in a future publication dedicated to the CC­(*P*) and CC­(*P*;*Q*) algorithms, as implemented in CCpy.

The above approach to handling term II in eq 19, in which we efficiently identify, sort, and compute the nonzero matrix elements of the ST, DT, and TT blocks of *H̅*_*N*_^(2)^, results in the possibility of speeding up CC­(*P*) calculations by factors of (*D*/*d*) for the ST and DT blocks and (*D*/*d*)^2^ for the TT block relative to their full CCSDT counterparts, where we use the same notation as in our discussion of term (I), in which *D* defines the number of all triply excited determinants and *d* is the number of triples included in the *P* space. Combined with the substantial savings offered by our way of handling term (I) discussed above, we obtain a highly efficient algorithm for constructing and solving the CC­(*P*) amplitude equations, even when the triply excited determinants included in the *P* space do not form a continuous manifold. As already alluded to above, similar savings in the computational effort apply to the companion left-eigenstate CC­(*P*) system based on eq 10, needed to obtain the deexcitation operator Λ^(*P*)^ = Λ_1_ + Λ_2_ + Λ_3_^(*P*)^, in which the subset of triples entering Λ_3_^(*P*)^ is the same as that defining *T*_3_^(*P*)^.

We end this appendix by illustrating the computational benefits offered by our CC­(*P*;*Q*) algorithm in CCpy, especially its key CC­(*P*) part discussed above, by comparing the CPU times needed to solve the CC­(*P*) amplitude and left-eigenstate equations and to form the noniterative δ­(*P*;*Q*) corrections with those required by the parent full CCSDT approach for the singlet ground state of cyclobutadiene, as described by the cc-pVDZ and cc-pVTZ basis sets, at the challenging TS structure along its automerization coordinate corresponding in eq 2 to λ = 1, where *T*_3_ correlations become large, nonperturbative, and difficult to capture. All the CPU times reported below correspond to single-core runs on a PowerEdge R940 server from Dell equipped with Intel Xeon Gold 6252 2.1 GHz processor boards. The CC­(*P*) and CC­(*P*;*Q*) calculations using the lists of triply excited determinants included in the underlying *P* spaces generated by CIPSI were performed with CCpy, whereas the parent CCSDT computations were carried out using our highly efficient, fully vectorized, CCSDT codes available in GAMESS. As in all of the CIPSI-driven CC­(*P*) and CC­(*P*;*Q*) computations discussed in the main text, no advantage of the *D*_4h_ symmetry of the TS structure of cyclobutadiene or its *D*_2h_ Abelian subgroup was taken in any of the post-RHF calculations. In presenting the timings characterizing the CC­(*P*) and CC­(*P*;*Q*) computations, the computational times associated with the execution of the integral, RHF, and integral transformation and sorting routines are ignored.

Our first set of timings involves the calculations using the cc-pVDZ basis set. In this case, we needed 176.5 CPU minutes and 30 iterations to converge the CCSDT energy of the cyclobutadiene TS species to 10^–7^ hartree. The corresponding CC­(*P*) amplitude equations using 0.5% of triply excited determinants in the *P* space identified by the CIPSI run with *N*_det(in)_ = 250,000, which, as shown in Table 1, after correcting for the remaining triples not included in the *P* space via the CC­(*P*;*Q*) correction δ­(*P*;*Q*), reproduce the CCSDT energy to within 0.458 millihartree, needed only 2.3 CPU minutes and 19 iterations to converge. This is a speedup by a factor of 77 compared to CCSDT. The combined time spent on solving the CC­(*P*) amplitude equations and the associated left-eigenstate problem that provides the information used to determine the $l_{K}(P)$ coefficients multiplying moments $M_{K}\left(P\right)$ in eq 7 for δ­(*P*;*Q*) was 4.1 CPU minutes, and the time needed to determine the noniterative δ­(*P*;*Q*) correction was 0.9 CPU minutes, which are again considerable savings in the computational effort compared to CCSDT. The latter time is somewhat longer than the 0.5 CPU minutes needed to calculate the noniterative triples correction of CR-CC­(2,3), since moments

A15 $$M_{abc}^{ijk}\left(P\right)=\left\langle \Phi_{ijk}^{abc}\right|{\mathrm{H̅}}^{\left(P\right)}\left|\Phi \right\rangle ,\left|\Phi_{ijk}^{abc}\right\rangle \in H^{\left(Q\right)}$$

and the associated coefficients

A16 $$l_{ijk}^{abc}\left(P\right)=\left\langle \Phi \right|\left(1+\Lambda^{\left(P\right)}\right){\mathrm{H̅}}^{\left(P\right)}\left|\Phi_{ijk}^{abc}\right\rangle /D_{abc}^{ijk}(P),$$

where *D*_*abc*_^*ijk*^(*P*) = *E* ^(*P*)^ − ⟨Φ_*ijk*_^*abc*^|*H̅* ^(*P*)^|Φ_*ijk*_^*abc*^⟩, used to compute the δ­(*P*;*Q*) correction when there are some triply excited determinants in the *P* space, engage the *T*_1_, *T*_2_, and *T*_3_^(*P*)^ components of the cluster operator *T* ^(*P*)^ in constructing the similarity-transformed Hamiltonian *H̅* ^(*P*)^, as opposed to only *T*_1_ and *T*_2_ obtained with CCSD used to construct $M_{abc}^{ijk}\left(2\right)$ , eq 26, in CR-CC­(2,3), and the three-body component of the deexcitation operator Λ^(*P*)^, not used by CR-CC­(2,3) either, but it is still almost 200 times shorter than the time needed to converge CCSDT and 6 times shorter than the timing characterizing a single CCSDT iteration. In fact, the combined time required to solve the CC­(*P*) amplitude equations and the corresponding left-eigenstate problem and to construct the CC­(*P*;*Q*) correction δ­(*P*;*Q*) turned out to be shorter than that associated with a single CCSDT iteration too.

The timings characterizing the CC­(*P*) and CC­(*P*;*Q*) computations for the cyclobutadiene TS species using the cc-pVTZ basis are similarly encouraging. The CCSDT amplitude equations converged to 10^–7^ hartree in 7,356.9 CPU minutes, requiring 30 iterations. The analogous CC­(*P*) calculations using 0.1% of triply excited determinants in the *P* space identified by the CIPSI run with *N*_det(in)_ = 1,000,000, which, after accounting for the remaining triples outside the *P* space using the CC­(*P*;*Q*) correction δ­(*P*;*Q*), reproduce the CCSDT energy to within 0.591 millihartree (cf. Table 4), needed 19 iterations and only 122.5 CPU minutes to converge, speeding up the CCSDT computations by a factor of 60. The cumulative time required to solve the CC­(*P*) amplitude equations and the associated left-eigenstate problem, of 232.4 CPU minutes, and the 8.3 CPU minutes used to calculate the δ­(*P*;*Q*) correction represent substantial savings in the computational effort compared to CCSDT as well. Once again, the 8.3 CPU minutes spent on constructing the noniterative correction δ­(*P*;*Q*) is more than the 5.2 CPU minutes needed to determine the triples correction of CR-CC­(2,3), which is a consequence of using moments $M_{abc}^{ijk}\left(P\right)$ , eq 22, involving *T*_3_^(*P*)^, in addition to *T*_1_ and *T*_2_, instead of the less demanding $M_{abc}^{ijk}\left(2\right)$ using only *T*_1_ and *T*_2_ obtained with CCSD exploited in CR-CC­(2,3), and coefficients $l_{ijk}^{abc}\left(P\right)$ , eq 23, engaging *H̅* ^(*P*)^ and Λ_3_^(*P*)^, but the overall message that both the CC­(*P*) computations and the noniterative steps of CC­(*P*;*Q*) are orders of magnitude less expensive than the parent CCSDT runs remains. Again, the cumulative time associated with solving the CC­(*P*) amplitude and left-eigenstate equations and to determine the CC­(*P*;*Q*) correction δ­(*P*;*Q*) turned out to be somewhat shorter than the time spent on a single CCSDT iteration.

It should be clear from the above analysis that our CC­(*P*;*Q*) codes in CCpy aimed at accurately approximating the CCSDT energetics using small fractions of triples in the underlying *P* spaces, which were identified in this work with CIPSI, can offer significant savings in the computational effort compared to full CCSDT, even when *T*_3_ effects and electronic quasidegeneracies become substantial and noniterative corrections to CCSD struggle. We have also demonstrated that a significant part of this success is due to our novel approach to implementing the CC­(*P*) equations, which can efficiently handle small but generally spotty subsets of triply excited determinants in the underlying *P* spaces. We will continue examining if our CC­(*P*;*Q*) codes in CCpy, especially the routines that construct and solve the CC­(*P*) equations, can achieve even greater speedups compared to CCSDT.

**A1 tbl7:** Programmable Expressions for the Matrix Elements of *H̅*_*N*_^(2)^ in the ST, DT, and TT Sectors Entering Term (II) of Equation A8, Excluding the Contributions in the TT Block due to the Three-Body Component of the *H̅*_*N*_^(2)^ Operator, Organized According to the Possible μ*p*-*νh* Differences Between Bra and Ket Determinants, as Defined in the Appendix

Matrix Element	μ*p*-ν*h* Difference	Expression^a^	Index Constraint^b^
$\left\langle \Phi_{i}^{a}\right|{\mathrm{H̅}}_{N}^{\left(2\right)}\left|\Phi_{lmn}^{def}\right\rangle$ ^c^	2*p*-2*h*	Al/mnAd/efh̅mn efδliδad	|*S*_*h*_| = |*S*_*p*_| = 1

$\left\langle \Phi_{ij}^{ab}\right|{\mathrm{H̅}}_{N}^{\left(2\right)}\left|\Phi_{lmn}^{def}\right\rangle$ ^d^	1*p*-1*h*	An/lmAf/deh̅n fδliδmjδadδbe	|*S*_*h*_| = |*S*_*p*_| = 2
	1*p*-2*h*	−AijAl/mnAf/deh̅mn jfδliδadδbe	|*S*_*h*_| = 1, |*S*_*p*_| = 2
	2*p*-1*h*	AabAd/efAn/lmh̅bn efδliδadδmj	|*S*_*h*_| = 2, |*S*_*p*_| = 1

$\left\langle \Phi_{ijk}^{abc}\right|{\mathrm{H̅}}_{N}^{\left(2\right)}\left|\Phi_{lmn}^{def}\right\rangle$ ^e^	0*p*-1*h*	−Ak/ijAn/lmh̅n kδadδbeδcfδliδmj	|*S*_*h*_| = 2, |*S*_*p*_| = 3
	1*p*-0*h*	Ac/abAf/deh̅c fδadδbeδliδmjδnk	|*S*_*h*_| = 3, |*S*_*p*_| = 2
	1*p*-1*h*	AijkAabcAl/mnAd/efh̅al idδnkδmjδbeδcf	|*S*_*h*_| = |*S*_*p*_| = 2
	0*p*-2*h*	Ak/ijAn/lmh̅lm ijδnkδadδbeδcf	|*S*_*h*_| = 1, |*S*_*p*_| = 3
	2*p*-0*h*	Ac/abAf/deh̅ab deδnkδliδmjδcf	|*S*_*h*_| = 3, |*S*_*p*_| = 1

a In the expressions reported in this column, δ_*p*_^*q*^ is the Kronecker delta and $A_{pq}$ = $A^{pq}$ = 1 − (*pq*), $A_{p/qr}$ = $A^{p/qr}$ = 1 − (*pq*) − (*pr*), and $A_{pqr}$ = $A^{pqr}$ = $A_{qr}A_{p/qr}$ = $A^{qr}A^{p/qr}$ = 1 – (*pq*) – (*pr*) – (*qr*) + (*pqr*) + (*prq*) are index antisymmetrizers, with (*pq*) designating the transposition of *p* and *q*.

b The relevant set intersections describing the hole and particle indices common to the bra and ket determinants are defined as *S*_*h*_ = {*i*,...} ∩ {*l*,...} and *S*_*p*_ = {*a*,...} ∩ {*d*,...}, respectively, with |*S*_*h*_| and |*S*_*p*_| representing the cardinal numbers of *S*_*h*_ and *S*_*p*_.

c The triply excited determinant specifying the ket assumes that the spin-orbital hole and particle indices are ordered such that *l* < *m* < *n* and *d* < *e* < *f*.

d The doubly and triply excited determinants specifying the bra and ket assume that the spin-orbital hole and particle indices are ordered such that *i* < *j*, *a* < *b*, *l* < *m* < *n*, and *d* < *e* < *f*.

e The triply excited determinants specifying the bra and ket assume that the spin-orbital hole and particle indices are ordered such that *i* < *j* < *k*, *a* < *b* < *c*, *l* < *m* < *n*, and *d* < *e* < *f*. Only the contributions due to the one- and two-body components of *H̅*_*N*_^(2)^ are considered. The contributions due to the three-body component of *H̅*_*N*_^(2)^ are embedded in the ${\mathrm{M̃}}_{abc}^{ijk}\left(2\right)$ quantity defined in eq 20 (see the Appendix for further details).

## Supplementary Material



## Notes

Results of the active-orbital-based CCSDt and CC­(t;3) calculations for the lowest singlet and triplet states of cyclobutadiene used to construct the CCSDt and CC­(t;3) potentials presented in Figure 6 (PDF)

## Notes

We dedicate this article to the memory of the late Professor John F. Stanton, whose seminal contributions to quantum chemistry, especially coupled-cluster theory, have inspired us over the years.
