# Supplementary material for: The Singlet–Triplet Gap of Cyclobutadiene: The CIPSI-Driven CC(P;Q) Study
Source: J Phys Chem A. 2025 Dec 9;129(50):11749–80. doi: 10.1021/acs.jpca.5c07572 (PMC12720243; doi:10.1021/acs.jpca.5c07572)
Supplement: Supplementary file 1 [file jp5c07572_si_001.pdf]

Supporting Information for “The Singlet–Triplet Gap of Cyclobutadiene: The CIPSI-Driven CC(P;Q) Study”

Swati S. Priyadarsini,[1] Karthik Gururangan,[1] Jun Shen,[1] and Piotr Piecuch[1,2,a)]
[1)] *Department of Chemistry, Michigan State University, East Lansing, Michigan 48824, USA*
[2)] *Department of Physics and Astronomy, Michigan State University, East Lansing, Michigan 48824, USA*

(Dated: 10 November 2025)

In Tables S1 – S3 of this Supporting Information document, we report the results of the active-orbital-based CCSDt and CC(t;3) calculations for the lowest singlet and triplet states of cyclobutadiene, as described by the cc-pVDZ basis set, and the gap between them along its D_{2h}-symmetric automerization coordinate that were used to prepare Figure 6 of the main text.

[a)] Corresponding author; e-mail: piecuch@chemistry.msu.edu.

TABLE S1. Results of the Active-Orbital-Based CCSDt and CC(t;3) Calculations for the Lowest Singlet State of Cyclobutadiene, as Described by the cc-pVDZ Basis Set, at Selected Values of Parameter λ Defining the Automerization Coordinate via the Interpolation Formula Given by Eq. (2) in the Main Text.

λ	CCSDt[a,b]	CC(t;3)[a,b]
0	20.785	−0.137
0.2	20.792	−0.192
0.4	20.745	−0.258
0.6	20.605	−0.324
0.8	20.365	−0.323
1	20.274	0.071

[a] The CCSDt and CC(t;3) energies are reported as errors relative to CCSDT in millihartree. The total CCSDT energies at $\lambda = 0$, 0.2, 0.4, 0.6, 0.8, and 1 are −154.244157, −154.242922, −154.240027, −154.236079, −154.232439, and −154.232002 hartree, respectively.

[b] The active space defining the subset of triply excited determinants included in the CCSDt calculations preceding the determination of the noniterative CC(t;3) corrections consisted of the highest occupied and lowest unoccupied RHF orbitals correlating with the valence e_g shell of the square TS structure of cyclobutadiene. In the language of the CC(P) and CC(P;Q) frameworks, the P space obtained in this way contained 1.5% of the $S_z = 0$ triples of the $A_g(D_{2h})$ symmetry involved in the parent full CCSDT work.

TABLE S2. Results of the Active-Orbital-Based CCSDt and CC(t;3) Calculations for the Lowest Triplet State of Cyclobutadiene, as Described by the cc-pVDZ Basis Set, at Selected Values of Parameter λ Defining the Automerization Coordinate via the Interpolation Formula Given by Eq. (2) in the Main Text.

λ	CCSDt[a,b]	CC(t;3)[a,b]
0	21.129	−0.400
0.2	20.962	−0.398
0.4	20.821	−0.396
0.6	20.707	−0.395
0.8	20.619	−0.393
1	20.546	−0.393

[a] The CCSDt and CC(t;3) energies are reported as errors relative to CCSDT in millihartree. The total CCSDT energies at $\lambda = 0$, 0.2, 0.4, 0.6, 0.8, and 1 are −154.195389, −154.205779, −154.213867, −154.219672, −154.223190, and −154.224380 hartree, respectively.

[b] The active space defining the subset of triply excited determinants included in the CCSDt calculations preceding the determination of the noniterative CC(t;3) corrections consisted of the two singly occupied ROHF orbitals correlating with the valence e_g shell of the square TS structure of cyclobutadiene. In the language of the CC(P) and CC(P;Q) frameworks, the P space obtained in this way contained 1.1% of the $S_z = 1$ triples of the $B_{1g}(D_{2h})$ symmetry involved in the parent full CCSDT work.

TABLE S3. Results of the Active-Orbital-Based CCSDt and CC(t;3) Calculations for the Singlet–Triplet Gaps $\Delta E_{\mathrm{S-T}} = E_{\mathrm{S}} - E_{\mathrm{T}}$ Characterizing Cyclobutadiene, as Described by the cc-pVDZ Basis Set, at Selected Values of Parameter λ Defining the Automerization Coordinate via the Interpolation Formula Given by Eq. (2) in the Main Text.

λ	CCSDt[a,b]	CC(t;3)[a,b]
0	−0.216	0.165
0.2	−0.107	0.129
0.4	−0.048	0.087
0.6	−0.064	0.044
0.8	−0.159	0.044
1	−0.171	0.291

[a] The CCSDt and CC(t;3) singlet–triplet gaps, computed using the energies of the lowest singlet and triplet states provided in Tables S1 and S2, are reported as errors relative to CCSDT in kcal/mol. The CCSDT singlet–triplet gap values at $\lambda = 0$, 0.2, 0.4, 0.6, 0.8, and 1 are −30.603, −23.308, −16.416, −10.295, −5.804, and −4.783 kcal/mol, respectively.
